# Direct glucosone-based synthesis and HILIC-ESI-MS/MS characterization of *N*-terminal fructosylated valine and valylhistidine for validation of enzymatic HbA_1c_ assays in the diagnosis of *diabetes mellitus*

**DOI:** 10.1007/s00216-019-02186-2

**Published:** 2019-11-22

**Authors:** Christoph Gerke, Monika Buchholz, Holger Müller, Reinhard Meusinger, Matthias Grimmler, Erwin Metzmann

**Affiliations:** 1 Hochschule Fresenius, University of Applied Sciences, Limburger Straße 2, 65510 Idstein, Germany; 2 Present Address: Department of Chemistry in Pharmaceutical Sciences, Faculty of Pharmacy, Complutense University of Madrid, Plaza de Ramón y Cajal s/n, 28040 Madrid, Spain; 3 DiaSys Diagnostic Systems GmbH, Alte Straße 9, 65558 Holzheim, Germany; 4 Clemens-Schöpf Institute of Organic Chemistry and Biochemistry, University of Technology Darmstadt, Alarich-Weiss-Straße 4, 64287 Darmstadt, Germany

**Keywords:** Fructosylated hemoglobin, Fructosamines, HbA_1c_ quantification, FPOX assay, *Diabetes mellitus*, HILIC-ESI-MS/MS

## Abstract

Naturally occurring fructosamines are of high clinical significance due to their potential use in *diabetes mellitus* monitoring (quantification of fructosylated hemoglobin, HbA_1c_) or for the investigation of their reactivity in consecutive reactions and harmfulness towards the organism. Here we report the specific synthesis of the fructosylated dipeptide l-valyl-l-histidine (Fru-Val-His) and fructosylated l-valine (Fru-Val). Both are basic tools for the development and validation of enzymatic HbA_1c_ assays. The two fructosamine derivatives were synthesized via a protected glucosone intermediate which was coupled to the primary amine of Val or Val-His, performing a reductive amination reaction. Overall yields starting from fructose were 36% and 34% for Fru-Val and Fru-Val-His, respectively. Both compounds were achieved in purities > 90%. A HILIC-ESI-MS/MS method was developed for routine analysis of the synthesized fructosamines, including starting materials and intermediates. The presented method provides a well-defined and efficient synthesis protocol with purification steps and characterization of the desired products. The functionality of the fructosylated dipeptide has been thoroughly tested in an enzymatic HbA_1c_ assay, showing its concentration-dependent oxidative degradation by fructosyl-peptide oxidases (FPOX).

**Electronic supplementary material:**

The online version of this article (10.1007/s00216-019-02186-2) contains supplementary material, which is available to authorized users.

**Figure:**
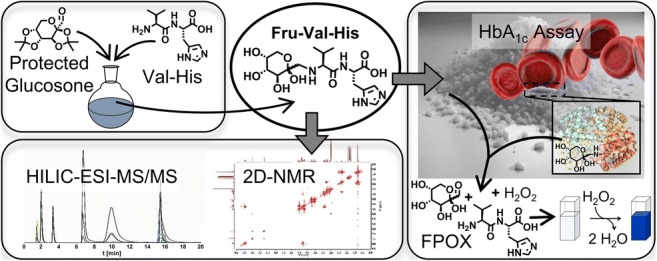
Graphical abstract

## Introduction

*Diabetes mellitus* is one of the leading medical threats with 451 million affected patients worldwide in 2017 and approximately 693 million patients expected in 2045. Global healthcare spending of 850 billion USD was associated with the disease in 2017 [1]. Because there is still no cure available, early diagnosis and monitoring of the disease are essential to avoid and manage diabetic complications and to reduce the economic burden on societies. The most prominent characteristic of *diabetes mellitus* is the excessive circulation of d-glucose (Glc) in the blood (hyperglycemia). Glc in the bloodstream undergoes spontaneous, non-enzymatic condensation with primary amino groups of proteins forming an aldimine which subsequently rearranges to a fructosamine via the Amadori rearrangement [2].

In recent years, quantification of the fructosamine formed by the reaction of Glc with the *N*-terminus of the hemoglobin β-chain (fructosylated hemoglobin) has been established as an essential parameter in the management and diagnosis of diabetes. Fructosylated hemoglobin is referred to as “HbA_1c_” and its concentration is clinically reported in relation to total hemoglobin as a percentage or as a molar ratio (mmol/mol). Concentration levels above 6.5% or 48 mmol/mol HbA_1c_ are used as a threshold to diagnose and manage *diabetes mellitu*s [3]. HbA_1c_ quantification has been standardized by the introduction of an IFCC (The International Federation of Clinical Chemistry and Laboratory Medicine)-approved reference method based on peptide mapping after enzymatic digestion by electrospray mass spectrometry (ESI-MS) and capillary electrophoresis of the fructosylated *N*-terminal hexapeptide of the hemoglobin β-chain [4, 5]. Chromatographic procedures and immunoassays are the most common methods for the determination of the relative HbA_1c_ concentration in clinical laboratories. However, increasing requirements on specificity, precision, throughput, and cost-effectiveness have led to the development of several new assay methods for monitoring HbA_1c_. Some of these methods are based on the enzymatic quantification of HbA_1c_ [6–9]. The enzymatic methods are mainly facilitated by proteolytic digestion of hemoglobin and subsequent specific cleavage of fructosamines by fructosyl-amino acid oxidases or fructosyl-peptide oxidases generating hydrogen peroxide which can be measured photometrically by state-of-the-art procedures, mainly based on the oxidation of a leuco dye [6–8]. The substrates of these oxidases are fructosylated l-valine (Fru-Val) or fructosylated l-valyl-l-histidine (Fru-Val-His), from which primarily the fructosylated dipeptide is used in commercially available enzymatic assays [10–12]. It is of utmost importance to have access to well-defined substrates of these specific oxidases to assess specificity and reaction conditions and to reach a high level of commutability of HbA_1c_ assays. The unsatisfying situation towards standardization of enzymatic HbA_1c_ assays is also reflected by the commercial unavailability of suitable fructosylated dipeptides. Specifically, the syntheses of the two fructosylated compounds have been only described by the working groups of Dixon [13] (Fru-Val-His) and by Keil [14] (Fru-Val) without providing data on composition and purity to confirm the structure of the compound. Although the synthesis and characterization of many fructosylated amino acids and peptides have been reported in literature, the majority study kinetics of their formation, specific analytical data, or the reported synthetic approaches lead to unsatisfying yields or purities [13–17]. Only few information about their isolation in good yields and purities has been reported, using either protected sugars as presented by the groups of Hoffmann and Horvat [18–20] or zinc halide catalysts as by the groups of Harohally and Norin [21–23]. The synthetic approach that mimics the natural reaction of free Glc with a primary amine of a protein occurring in the organism is currently still widely used by working groups studying Amadori compounds or developing HbA_1c_ assays [24–28]. However, this approach usually leads to a complex, dark syrup-like mixture containing various side products from which the desired fructosamine is subsequently crystallized. Successful crystallization of the fructosylated product highly depends on the number of side products formed. Furthermore, even after crystallization, the desired fructosamine has to be habitually further purified. For this, it is difficult to achieve a reproducible and robust process with reasonable effort to obtain the pure product. Besides lack of suitable purification protocols, the efficiency of a full conversion of acyclic Glc can be seen as the major obstacle in obtaining suitable yields of the product even after long reaction times, since the reactive, acyclic Glc form is only present in extremely slight amounts of approximately 0.002% in solution [29].

Here we now present a direct approach using for the first time the finally targeted d-fructose (Fru) for the synthesis of the corresponding fructosamines Fru-Val and Fru-Val-His of HbA_1c_. Therefore, the used Fru intermediate is, on the one hand, functionalized with protecting groups on hydroxyl groups not taking part in the reaction, thereby preventing the formation of side products. On the other hand, a constantly present reactive aldehyde group at the future linkage position is introduced to ensure a higher conversion compared with the approach using unfunctionalized Glc. For routine analysis of the fructosamine derivatives, a HILIC-ESI-MS/MS method is developed which also includes all starting materials as well as intermediates from the synthetic pathway. Thereby, the synthesized fructosamines are able to be unambiguously characterized and the presence of remaining starting materials can be determined. The finally obtained synthetic Fru-Val-His is further applied in enzymatic assays for HbA_1c_ quantification to investigate and ensure its functional use as substrates for FPOX enzymes.

## Materials and methods

### Materials

l-Valine (≥ 98.5%), l-histidine (≥ 99%), trifluoroacetic acid (TFA) (≥ 99.9%), potassium carbonate (≥ 99%), methanol (≥ 99.9%), ethanol (96%), diethyl ether (with BHT as inhibitor, ≥ 99.5%), cyclohexane (≥ 99.5%), dichloromethane (DCM) (> 99.5%), dimethyl sulfoxide (DMSO) (≥ 99.5%), sulfuric acid (96%), hydrochloric acid (HCl) (37%), acetonitrile (ACN) (≥ 99.98%), and formic acid (≥ 98%) as well as cation exchange resin DOWEX 50W X2 200–400 mesh in H^+^ form and silica gel 60 0.04–0.063 mm (230–400 mesh) were purchased from Carl Roth GmbH & Co. KG (Karlsruhe, Germany). d-(-)-Fructose (99%), phosphomolybdic acid hydrate (≥ 99.99%), oxalyl chloride (98%), sodium hydride (95%), triethylamine (99%), and ammonium formate (≥ 99.0%) were purchased from Sigma-Aldrich Inc. (St. Louis, USA). Sodium sulfate (99%), ninhydrin (> 98%), ammonium chloride (≥ 99.5%), and sodium cyanoborohydride (95%) were purchased from Merck KGaA (Darmstadt, Germany). Dipeptide l-valyl-l-histidine was purchased from Bachem AG (Bubendorf, Switzerland). Sea sand, activated charcoal, and sodium bicarbonate (99.5%) were all purchased from Riedel-de-Haën AG (Seelze, Germany). Thin-layer plates with 0.2-mm silica gel and fluorescent indicator with a size of 40 × 80 mm were bought from Macherey-Nagel GmbH & Co. KG (Düren, Germany). Enzymatic assay of HbA_1c_ (containing fructosyl-peptide oxidase (FPOX), horseradish peroxidase (HRP), and leuco form DA67 of methylene blue reagent core components) and HbA_1c_ calibrator, controls, and hemolyzing solution were provided by DiaSys Diagnostic Systems GmbH (Holzheim, Germany). Ultra-pure grade water type 1 was obtained from a Simplicity System Unit with SimFilter (0.05 μm) final filter from Merck Millipore (Darmstadt, Germany).

### Synthetic procedures and analytical data of synthesized compounds

2,3:4,5 di-*O*-Isopropylidene-β-d-fructopyranose (*ipr*Fru, **1**) was synthesized according to a published protocol from Pacsu et al. [30], using 15.1 g (84 mmol) d-fructose. The amounts of concentrated sulfuric acid (12 mL) as well as acetone (300 mL) were scaled down linearly for the used 15.1 g of d-fructose, taking the applied amounts of Pacsu et al. into account. 11.85 g (45.5 mmol) of *ipr*Fru **1** was obtained, corresponding to a yield of 54%. ^1^H NMR (500 MHz, CDCl_3_): *δ* 4.59 (dd, *J* = 7.9, 2.6 Hz, 1H), 4.32 (d, *J* = 2.6 Hz, 1H), 4.22 (ddd, *J* = 7.9, 2.0, 0.7 Hz, 1H), 3.90 (dd, *J* = 13.0, 2.0 Hz, 1H), 3.76 (dd, *J* = 13.0, 0.5 Hz, 2H), 3.69 (dd, *J* = 11.7, 8.4 Hz, 1H), 3.64 (dd, *J* = 11.7, 4.4 Hz, 1H), 2.06 ppm (dd, *J* = 8.5, 4.7 Hz, 1H). For annotation of protons, see Electronic Supplementary Material (ESM). ^13^C NMR (126 MHz, CDCl_3_): *δ* 109.27, 108.71, 103.26, 71.24, 70.99, 70.26, 65.79, 61.49, 26.65, 25.96, 25.53, 24.17 ppm. For annotation of carbon nuclei, see ESM. Signal assignment of NMR data was achieved by 2D-NMR measurements. M. p.: 96–97 °C. FT-IR (KBr disk): *ῦ* 3306, 2985, 2938, 1245, 1106, 1067 cm^−1^. HRMS for C_12_H_20_O_6_ (exact monoisotopic mass 260.1260 g/mol): [M+H]^+^ calcd. 261.1333; found 261.1334; mass accuracy 0.38 ppm.

2,3:4,5-di-*O*-Isopropylidene-aldehydo-β-d-arabinohexos-2-ulo-2,6-pyranose (*ipr*Glu, **2**) was synthesized performing a Swern oxidation adapted from the published procedure by the group of Hoffmann [18]. Prior to the reaction, instruments as well as solvents were freshly dried. 1.43 g (11.3 mmol, 1.5 equiv) oxalyl chloride was added to 35 mL DCM and cooled down to − 78 °C using a cooling bath with acetone and dried ice. After reaching the temperature, 1.76 g (22.5 mmol, 3 equiv) DMSO in 5 mL DCM was added to the solution within 5 min and the mixture was stirred for 20 min. Subsequently, 1.95 g (7.5 mmol) *ipr*Fru **1** dissolved in 5 mL DCM was added within 10 min and the mixture was stirred for an additional 60 min at − 78 °C. 3.42 g (33.8 mmol, 4.5 equiv) triethylamine (TEA) was added and stirred for 15 min at − 78 °C before the reaction mixture was allowed to reach room temperature. The reaction was performed under nitrogen atmosphere. The product was purified by washing the organic reaction mixture three times with 40 mL of water. Product in the separated aqueous phase was extracted four times by 40 mL DCM and the organic phases were combined. The organic phase was washed once with 60 mL of aqueous ammonium chloride (0.25 M) solution and twice with 60 mL saturated sodium bicarbonate solution. The washed organic phase was dried with sodium sulfate and the DCM was removed under reduced pressure, giving a yellow oil as crude product. Column chromatography (solvent mixture of diethyl ether and cyclohexane in a ratio of 50/50 (v/v)) afforded 1.39 g (5.38 mmol) of *ipr*Glu **2**, corresponding to a yield of 72%. Complex spectra from ^1^H as well as ^13^C NMR measurement were obtained because of the presence of *ipr*Glu as aldehyde and in its hydrate form in solution. FT-IR (film on NaCl): *ῦ* 2991, 2935, 1749, 1252, 1214, 1067 cm^−1^.

As further purification step as well as to clearly characterize the *ipr*Glu, it was converted into its hydrate form, following a procedure from Stefanowicz and co-workers [31]. A twofold amount of water was added to the oily crude *ipr*Glu. The mixture was stored in a fridge overnight, whereupon white crystals were formed. ^1^H NMR for the *ipr*Glu hydrate (500 MHz, DMSO-d_6_): *δ* 5.69 (d, *J* = 6.8 Hz, 1H), 5.62 (d, *J* = 6.4 Hz, 1H), 4.68 (t, *J* = 6.6 Hz, 1H), 4.54 (dd, *J* = 8.0, 2.6 Hz, 1H), 4.33 (d, *J* = 2.8 Hz, 1H), 4.20 (dd, *J* = 8.0, 1.5, 1H), 3.72 (dd, *J* = 13.1, 1.9 Hz, 1H), 3.57 (d, *J* = 13.0 Hz, 1H), 1.44 (s, 3H), 1.36 (s, 6H), 1.27 ppm (s, 3H). For annotation of protons, see ESM. ^13^C NMR for the *ipr*Glu hydrate (126 MHz, DMSO-d_6_): *δ* 108.01, 107.82, 103.52, 89.32, 70.14, 69.84, 69.49, 60.15, 26.47, 25.57, 25.53, 23.99 ppm. For annotation of carbon nuclei, see ESM. Signal assignment of NMR data was achieved by 2D-NMR measurements. M. p.: 82–84 °C (Lit. 71–72 °C [31]). FT-IR (KBr disk): 3399, 2985, 2943, 1244, 1220, 1075 cm^−1^. HRMS for C_12_H_18_O_6_ (exact monoisotopic mass 258.1103 g/mol): [M+NH_4_]^+^ calcd. 276.1442; found 276.1447; mass accuracy 1.81 ppm. HRMS for *ipr*Glu hydrate C_12_H_20_O_7_ (exact monoisotopic mass 276.1209 g/mol): [M+NH_4_]^+^ calcd. 294.1547; found 294.1553; mass accuracy 2.04 ppm.

Protected fructosylated Val (*ipr*Fru-Val, **3a**) and Val-His (*ipr*Fru-Val-His, **3b**) were synthesized performing a reductive amination of *ipr*Glu **2** and the primary amine of Val or Val-His, respectively, adapting the protocol from the group of Walton [32]. 0.7 mmol of Val or Val-His (see Table 1 for exact amounts) and 386.8 mg (1.4 mmol, 2 equiv) *ipr*Glu **2** hydrate were dissolved in a 10-mL mixture of water and methanol in a ratio of 1/1 (v/v) and stirred for 4 h at 70 °C. Subsequently, 88 mg (1.4 mmol) sodium cyanoborohydride dissolved in 2.5 mL water was added to the reaction mixture and stirred for an additional 7 h at 70 °C. After completion, 1 mL aqueous HCl solution (35%) and 4 mL water were added and the mixture stirred for 1 h to quench the reaction. An additional 5 mL of water was added and the mixture was applied to a column packed with DOWEX 50W X2 strong cation exchange (SCX) resin. The column was washed with 100 mL of water and the product subsequently eluted by applying 150 mL of aqueous ammonia solution (0.1 M). Fractions were collected and analyzed by TLC. Fractions containing the aimed product were pooled and the solvent removed under reduced pressure using a rotary evaporator. For isolated yields of protected Fru-Val and Fru-Val-His, see Table 1.

**Table 1:** Amounts of Val and Val-His used during reductive amination and obtained yields of *ipr*Fru-Val **3a** and *ipr*Fru-Val-His **3b** after reductive amination as well as of the final products Fru-Val **4a** and Fru-Val-His **4b** after isopropylidene removal

Reductive amination							Isopropylidene removal			
Starting material			Product				Product			
	m (mg)	n (mmol)	**#**	m (mg)	n (mmol)	Yield (%)	**#**	m (mg)	n (mmol)	Yield (%)
Val	81.9	0.70	**3a**	244	0.68	97	**4a**	180	0.64	94
Val-His	178	0.70	**3b**	297	0.60	86	**4b**	250	0.60	quant.

Analytical data for *ipr*Fru-Val **3a**: ^1^H NMR (500 MHz, CDCl_3_): *δ* 4.52 (dd, *J* = 7.9, 2.5 Hz, 1H), 4.17 (dd, *J* = 7.9, 1.8 Hz, 1H), 4.02 (d, *J* = 2.6 Hz, 1H), 3.81 (dd, *J* = 13.0, 2.0 Hz, 1H), 3.70 (d, *J* = 13.0 Hz, 1H), 3.05 (d, *J* = 12.0 Hz, 1H), 3.03 (d, *J* = 4.0 Hz, 1H), 2.64 (d, *J* = 12.0 Hz, 1H), 2.25–2.14 (m, 1H), 1.48 (s, 3H), 1.39 (s, 3H), 1.30 (s, 3H), 1.28 (s, 3H), 0.97 (d, *J* = 7.0 Hz, 3H), 0.90 ppm (d, *J* = 6.9 Hz, 3H). For annotation of protons, see ESM. ^13^C NMR (126 MHz, CDCl_3_): *δ* 174.11, 109.31, 108.79, 102.48, 72.49, 70.95, 70.29, 67.70, 61.49, 57.27, 31.06, 26.54, 26.10, 25.19, 24.23, 19.38, 17.92 ppm. For annotation of carbon nuclei, see ESM. Signal assignment of NMR data was achieved by 2D-NMR measurements. HRMS for C_17_H_29_NO_7_ (exact monoisotopic mass 359.1944 g/mol): [M+H]^+^ calcd. 360.2017; found 360.2018; mass accuracy 0.28 ppm.

Analytical data for *ipr*Fru-Val-His **3b**: ^1^H NMR (500 MHz, DMSO-d_6_): *δ* 7.94 (d, *J* = 7.1 Hz, 1H), 7.48 (s, 1H), 6.75 (s, 1H), 4.52 (dd, *J* = 7.9, 2.5 Hz, 1H), 4.36 (d, *J* = 2.6 Hz, 1H), 4.28 (q, *J* = 6.6 Hz, 1H), 4.19 (d, *J* = 7.8 Hz, 1H), 3.73 (d, *J* = 12.9 Hz, 1H), 3.54 (d, *J* = 13.0 Hz, 1H), 2.95 (dd, *J* = 14.8, 5.6 Hz, 1H), 2.88 (dd, *J* = 14.7, 7.0 Hz, 1H), 2.78 (d, *J* = 5.7 Hz, 1H), 2.74 (d, *J* = 12.4 Hz, 1H), 2.60 (d, *J* = 12.3 Hz, 1H), 1.82 (dq, J = 13.3, 6.7 Hz, 1H), 1.44 (s, 3H), 1.34 (s, 3H), 1.33 (s, 3H), 1.26 (s, 3H), 0.80 ppm (d, *J* = 6.8 Hz, 6H). For annotation of protons, see ESM. ^13^C NMR (126 MHz, DMSO-d_6_): *δ* 172.93, 172.31, 134.38, 107.93, 107.29, 103.00, 70.77, 70.16, 69.66, 67.82, 60.36, 53.97, 52.36, 30.74, 29.00, 26.28, 25.70, 25.30, 23.97, 18.99, 18.48 ppm. For annotation of carbon nuclei, see ESM. Signal assignment of NMR data was achieved by 2D-NMR measurements. HRMS for C_23_H_36_N_4_O_8_ (exact monoisotopic mass 496.2533 g/mol): [M+H]^+^ calcd. 497.2606; found 497.2609; mass accuracy 0.60 ppm.

Fructosylated Val (Fru-Val, **4a**) and Val-His (Fru-Val-His, **4b**) were obtained by removing the isopropylidene protecting groups under acidic conditions. Therefore, **3a** and **3b** were stirred in a mixture of trifluoroacetic acid (TFA) and water (95 vol% TFA) as described by the group of Hoffmann [18]. The reaction time was adapted to a total of 7 h for complete removal of the protecting groups, as monitored by direct ESI-MS analysis. The product was isolated by precipitation from cold diethyl ether. Fru-Val **4a** and Fru-Val-His **4b** were obtained in 94% and 100% yields, respectively. Isolated amounts are listed in Table 1.

Analytical data for Fru-Val **4a**: ^1^H NMR spectra were recorded in DMSO-d_6_. The measurement led to complex NMR spectra, containing signals for all four anomers present in solution (see ESM). However, the compound was clearly identified by 2D-NMR measurements and structural similarities to **3a**. ^1^H NMR (500 MHz, DMSO-d_6_): *δ* 3.93–3.46 (m, 5H), 3.20 (broad, 1H), 3.02–2.63 (m, 2H), 2.30–1.86 (m, 1H), 0.99–0.82 ppm (m, 6H). For annotation of protons, see ESM. HRMS for C_11_H_21_NO_7_ (exact monoisotopic mass 279.1318 g/mol): [M+H]^+^ calcd. 280.1390; found 280.1391; mass accuracy 0.36 ppm.

Analytical data for Fru-Val-His **4b**: ^1^H and ^13^C NMR spectra were recorded in DMSO-d_6_. Both measurements led to complex NMR spectra, containing signals for all four anomers present in solution (see ESM). However, the compound was clearly identified by 2D-NMR measurements and structural similarities to **3b**. ^1^H NMR (500 MHz, DMSO-d_6_): *δ* 9.01–8.99 (m, 1H), 8.97 (s, 1H), 7.50–7.35 (m, 1H), 4.78–4.60 (m, 1H), 4.03–3.96 (m, 1H), 3.95–3.91 (m, 1H), 3.83–3.75 (m, 1H), 3.70–3.62 (m, 1H), 3.61–3.48 (m, 1H), 3.44 (m, 1H, under overlaying signal of residue ethanol), 3.32–3.15 (m, 1H), 3.14–3.00 (m, 1H), 2.98–2.58 (m, 2H), 2.28–2.17 (m, 1H), 1.00–0.90 ppm (m, 6H). For annotation of protons, see ESM. ^13^C NMR (126 MHz, DMSO-d_6_): *δ* 171.4–171.2, 166.6–166.2, 133.9, 129.3–129.1, 117.7–116.90, 100.8, 99.2, 95.3, 83.2–81.6, 78.4, 74.9, 74.0, 70.3, 68.9, 68.7 64.9–64.3, 63.6–60.1, 51.5–51.30, 50.7, 50.0, 29.2–28.9, 26.2–26.0, 18.5–17.2 ppm. For annotation of carbon nuclei, see ESM. HRMS for C_17_H_28_N_4_O_8_ (exact monoisotopic mass 416.1907 g/mol): [M+H]^+^ calcd. 417.1979; found 417.1982; mass accuracy 0.72 ppm.

### HILIC-ESI-MS/MS method

All starting materials, intermediates and final products as well as l-histidine (His) were included in the HILIC-ESI-MS/MS method. For the HILIC-ESI-MS/MS analysis, an Agilent 1100 series HPLC system, consisting of a G1312A pump and a G1367A autosampler from Agilent Technologies Inc. (Santa Clara, USA), was used, coupled to a triple quadrupole API 2000 LC/MS/MS system with ESI source from AB SCIEX (Framingham, USA) as mass analyzer.

#### Determination of MS/MS parameters for routine MRM analysis

Each substance was separately injected in the MS system using a syringe pump with the flow rate of 10 μL/min. Single standard solutions for each substance with a concentration between 10 and 25 μg/mL were prepared. The substances were dissolved in a mixture of H_2_O and ACN with a ratio of 70/30 (v/v), containing 5 mM ammonium formate (NH_4_HCO_2_). All substances were measured in positive ESI mode. The molecular ion with the highest abundance in positive ESI was determined, performing a manual scan. After choosing the precursor ion for routine multiple reaction monitoring (MRM) analysis, the three most abundant transition ions after collision-induced dissociation (CID) as well as the collision energies (CE) and cell exit energies (CEX) for the used instrument were determined automatically by the system.

#### Development of HILIC HPLC method

For separation of the included compounds, a HILIC column (HALO Penta-HILIC; 2.1 mm × 150 mm, 2.7 μm, AMT Advanced Materials Technology Inc., Wilmington, USA) was used. The mobile phases A and B were mixtures of H_2_O/ACN in ratios of 95/5 (v/v) and 5/95 (v/v), respectively. Both eluents contained an additional 5 mM of NH_4_HCO_2_ and the pH was adjusted to 3.2 by formic acid. The optimal flow rate, starting composition of the eluents, and gradient were determined, performing various runs under different conditions. For all optimization experiments, a mix-standard in H_2_O/ACN in a ratio of 20/80 (v/v) containing all substances was used. Concentrations of the substances in the mix-standard used for optimization experiments varied from 6.5 to 138 μg/mL (see ESM). Ten microliters of mix-standard was injected for each run.

### Enzymatic assay

The enzymatic assay was performed manually with an UVIKON XL spectrophotometer from Goebel Instrumentelle Analytik GmbH (Au, Germany) and on an automated clinical chemistry analyzer Hitachi 917E from Roche Diagnostics (Basel, Switzerland). Donor EDTA blood samples from internal donation were used to assess the functionality of synthetic fructosamines. As stock solution, 11.2 mg Fru-Val-His **4b** was dissolved in 10 mL distilled water. Regarding the 3% of remaining Val-His, the concentration of Fru-Val-His was corrected to 10.9 mg per 10 mL. A total of five samples (S1–S5) were prepared for the assay: a blank which was hemolysis solution (S1), a blood hemolysate sample with an endogenous HbA_1c_ value of 0.185 mmol/L (20.22 mmol/mol HbA_1c_/Hb (IFCC) and 14.7 g/dL Hb) (S2) (blood sample and hemolysis solution in ratio of 1/20 (v/v)), and three blood hemolysate samples which were spiked with different Fru-Val-His concentrations (S3–S5). For the spiked sample containing the highest amount of Fru-Val-His (S5), 10 μL of the stock solution was added to 990 μL hemolyzed blood, resulting in a final Fru-Val-His concentration of 10.9 mg/L (26.1 μmol/L). 100 µL and 200 μL of S5 were used and the volume adjusted to a total of 300 μL using hemolyzed blood to obtain spiked samples S3 and S4, containing 3.62 mg/L (8.69 μmol/L) and 7.24 mg/L (17.4 μmol/L) of Fru-Val-His, respectively. These three spikes represent HbA_1c_ values of 0.359, 0.553, and 0.707 mmol/L, respectively, considering the endogenous HbA_1c_ value of the blood sample (0.185 mmol/L) and the 1/20 (v/v) dilution of the blood sample during automated measurements of HbA_1c_. Addition of the reagents and sample was performed manually using the UVIKON XL spectrophotometer and automatically by the automated clinical chemistry analyzer Hitachi 917E. The absorption at 660 nm was started to be measured directly after the addition of the sample, continuing the measurement for 10 min. The temperature was maintained at 37 °C. Proportionality between absorption and the amount of Fru-Val-His was examined, determining the total change in absorption from the starting until the endpoint of the measurement (ΔAbs) which was subsequently plotted against the Fru-Val-His **4b** concentration of the sample. ΔAbs of S1 was subtracted as a blank from ΔAbs of the other samples S2–S5.

## Results and discussion

### Synthesis of *ipr*Glu as reactive carbohydrate intermediate

**Scheme 1:**
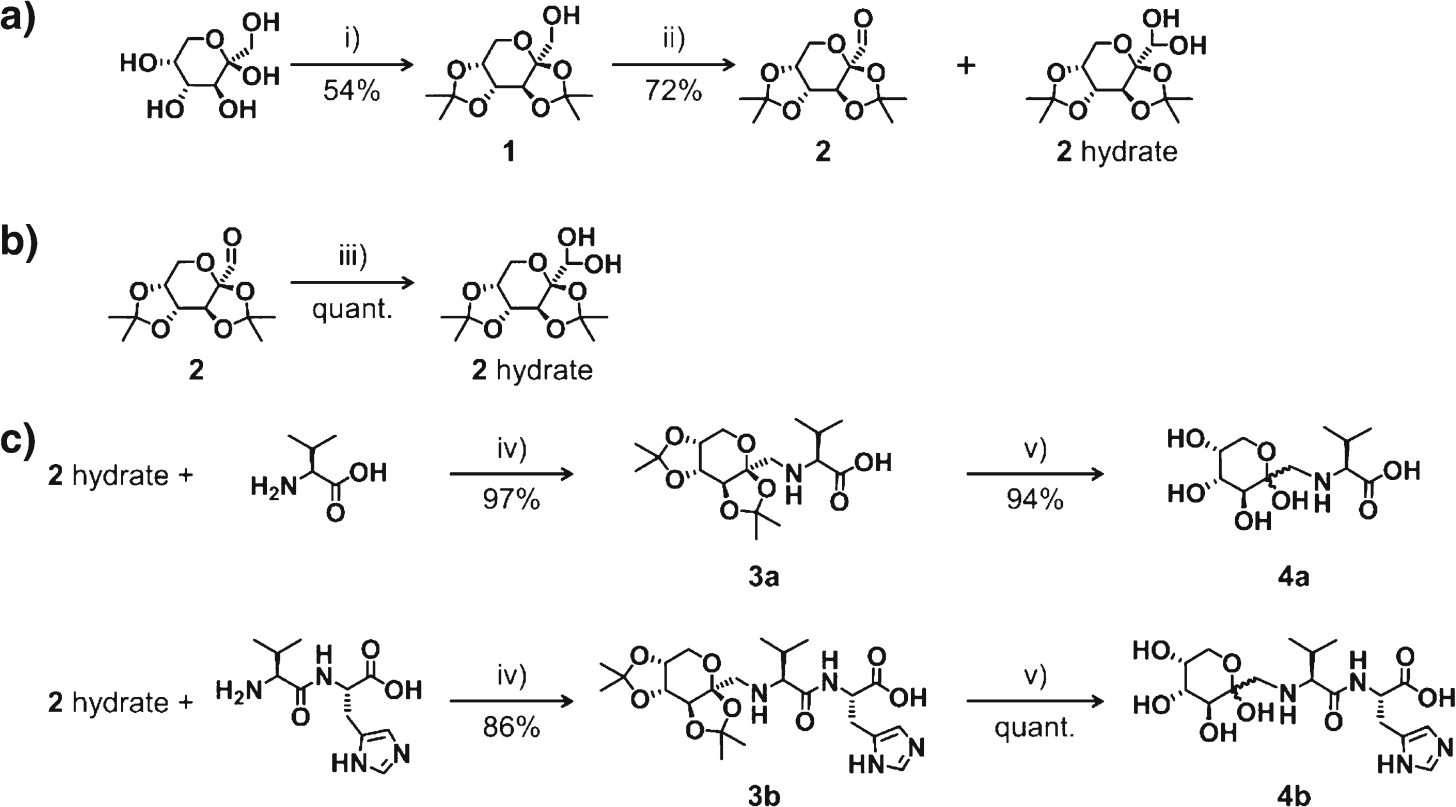
Synthesis of fructosamine derivatives Fru-Val **4a** and Fru-Val-His **4b** shown in c) via the protected carbohydrate intermediate *ipr*Glu **2** with its synthetic pathway as well as its hydrate form shown in **a** and **b**. Synthesis of *ipr*Glu **2** shown in **a**: i) acetone with sulfuric acid for 3 h below 0 °C; ii) oxalyl chloride and DMSO in DCM for 60 min at − 78 °C under N_2_ atmosphere, resulting in a mixture of *ipr*Glu **2** and its hydrate. The product **2** was quantitatively converted into its hydrate form as shown in **b**: iii) addition of water and crystallization of **2** hydrate at 4 °C overnight. Synthesis of the fructosamine derivatives Fru-Val **4a** and Fru-Val-His **4b** shown in **c**: iv) **2** hydrate and Val or Val-His, respectively, in water for 4 h at 70 °C followed by addition of sodium cyanoborohydride and reaction of additional 7 h at 70 °C; v) 95 vol% TFA in water for 7 h

To ensure a synthesis of the fructosamines Fru-Val and Fru-Val-His in sufficient yields with the formation of minor amounts of side products, an approach using a protected Fru intermediate was chosen. The hydroxyl groups on carbon atoms two to five (C2–C5) were therefore protected as isopropylidene ketals which are common protecting groups for diols [33–35], specifically for carbohydrates [36–38]. The isopropylidene ketals are introduced by the reaction with acetone under acidic condition. The reaction was performed following an established procedure from Pacsu and co-workers [30], resulting exclusively in the 2,3:4,5 di-*O*-isopropylidene-β-d-fructopyranose (*ipr*Fru, **1**) and not in the 1,2:4,5 isomer under the applied reaction conditions (see Scheme 1a). *ipr*Fru was obtained in a yield of 54% (batch size 84 mmol).

The remaining free hydroxyl moiety on carbon atom C1, which is the linkage position of the naturally occurring fructosamines, was subsequently oxidized to an aldehyde, allowing for the reaction with the primary amine of Val or Val-His, respectively. The oxidation was achieved by performing a Swern oxidation resulting in 2,3:4,5-di-*O*-isopropylidene-aldehydo-β-d-arabinohexos-2-ulo-2,6-pyranose which is isopropylidene ketal protected glucosone (*ipr*Glu, **2**). The synthesis was conducted following a procedure from the group of Hoffmann [18], and after purification by column chromatography, a yield of 72% (7.5 mmol batch size) *ipr*Glu was obtained. Due to ambiguous analytical data of *ipr*Glu, which is present as the aldehyde as well as its stable hydrate from, it was converted completely into its stable hydrate form under the addition of water, which subsequently crystallized as white solid [31]. Despite the fact that hydrates of carbonyls, the so-called *gem*-diols, are generally not stable and can therefore not be isolated, there are some exceptions [39–41]. In this case, the crystalline *gem*-diol structure was studied by Stefanowicz et al. [31], who also demonstrated that the hydrate converts back to the aldehyde in solution, therefore not hampering further reactions of the aldehyde group. Complete conversion of *ipr*Glu into its hydrate form was confirmed by NMR and IR spectroscopy (see ESM). The complete reaction pathway of *ipr*Glu as well as its hydrate form is shown in Scheme 1a.

### Synthesis of fructosamines Fru-Val and Fru-Val-His using *ipr*Glu

The resulting *ipr*Glu **2** can be coupled to the free primary amine of the amino acid Val or dipeptide Val-His with its aldehyde group on the C1 position, generating an aldimine which can be subsequently reduced to the desired fructosamine, as shown in Scheme 1b. The reductive amination was performed following a protocol from Walton et al. [32], using sodium cyanoborohydride (NaBH_3_CN) as reducing agent. Despite the fact that NaBH_3_CN does generally not reduce aldehydes [42, 43], for this reason being widely established as reducing agent in reductive amination, we clearly identified *ipr*Fru **1** by MS and NMR analyses as side product. We assume that the unique properties of the aldehyde of *ipr*Glu **2**, also responsible for the formation of its stable hydrate form, may be the reason for its reduction by the mild reducing agent NaBH_3_CN. We adapted the procedure from Walton et al. [32] by previously performing the reaction to the aldimine prior to the addition of the reducing agent. Therefore, *ipr*Glu **2** and the respective amino acid or dipeptide were stirred for 4 h at 70 °C in a mixture of water and methanol (ratio of 1/1 (v/v)), followed by the addition of NaBH_3_CN and an additional 7 h stirring at 70 °C. The reaction mixture was purified by strong cation exchange (SCX) chromatography, using a DOWEX 50W X2 resin, thereby removing excessive *ipr*Glu **2**. However, remaining Val or Val-His are assumed not to be removed by the performed purification and a full conversion was aimed by the adapted reaction procedure. Yields of 97% and 86% for *ipr*Fru-Val **3a** and *ipr*Fru-Val-His **3b** were achieved (0.7 mmol batch size), respectively. As final step, the protecting groups of the Fru residue were removed under acidic conditions by stirring the protected fructosamines in a solution of 95 vol% trifluoroacetic acid (TFA) in water. The reaction was monitored by direct ESI-MS analysis and complete removal of the isopropylidene ketal protecting groups was achieved after 7 h. The final products were isolated with almost quantitative yields (Table 1) by precipitation from cold diethyl ether and analyzed by HRMS, NMR (see ESM), and HILIC-ESI-MS/MS. The final fructosamines can adapt five different configurations in solution due to the mutarotation of the fructose moiety, which results in multiple signals in ^1^H and ^13^C NMR spectra (see ESM). From NMR data, the presence of mayor impurities was able to be excluded. Especially spectra from the protected fructosamines *ipr*Fru-Val **3a** and *ipr*Fru-Val-His **3b** solely showed signals for nuclei from the synthesized compounds in both ^1^H as well as in ^13^C NMR analysis, with all significant signals being able to be unambiguously assigned (see ESM). Due to the multiple signals in NMR data from the final, unprotected fructosamines Fru-Val **4a** and Fru-Val-His **4b**, purity determination becomes more demanding. However, all signals could be assigned by 2D-NMR experiments and by comparison with NMR data of their protected counterparts. Besides the signals from nuclei of the final products, solely signals from residue solvents diethyl ether, ethanol, and TFA were observed despite the application of high vacuum. In future studies, we aim to lyophilize the final products to fully remove residue solvents. HILIC-ESI-MS/MS data revealed the presence of very slight amounts of remaining starting material Val or Val-His in the final products. This is further discussed in the following section. Combining all analytical data obtained, purities of the final fructosamines Fru-Val **4a** and Fru-Val-His **4b** were determined all to be above 90%.

### HILIC-ESI-MS/MS method development for routine analysis of fructosamines Fru-Val and Fru-Val-His

**Fig. 1:**
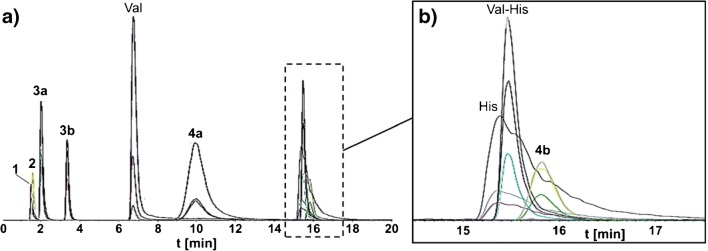
Selected ion chromatogram (SIC) of the developed HILIC-ESI-MS/MS method, display signals for the three selected transition ions for each compound: **a** SIC of the entire HILIC run with annotated signals, **b** offset of the SIC from minute 14.5 until 17.5, displaying signals of co-eluting compounds His, Val-His, and Fru-Val-His **4b**

For the routine analysis of synthesized fructosamine derivatives Fru-Val **4a** and Fru-Val-His **4b**, a HILIC-ESI-MS/MS method, with the tandem MS working in multiple reaction monitoring (MRM) mode, was developed. An MRM method was chosen instead of the commonly used product ion scan in routine analysis to further increase the sensitivity for the compounds of interest. Besides the final fructosamines, all starting materials and intermediates as well as His were included in the method. For a clear identification of the included substances, three specific transition ions from the most abundant precursor ion in positive ESI mode were determined. Furthermore, optimal parameters for collision-induced dissociation (CID) were determined. The precursor ions and the three determined transitions as well as collision energies (CE) and cell exit potentials (CXP) for each substance are listed in Table 2. Observed transition ions were typical for amino acids [44], peptides [45], carbohydrates [46], and fructosamines [15, 16] and proposed structures of transition ions are given in the ESM. Following the optimization of MRM mode parameters, a HILIC method including all 9 compounds was developed. Varied parameters were the flow rate, the starting composition of the eluent, and the gradient, aiming highest possible resolution for included compounds as well as short run times for high-throughput routine analysis. The flow rate was set to 0.25 mL/min and as starting eluent composition, a mixture of 15% A and 85% B was applied. After 5 min applying 15% A, the amount of eluent A was linearly increased to 25% within the next 6 min (from minute 5 until 11 of the run). The 25% of eluent A held for an additional 9 min (from minute 11 until 20 of the run), before stopping the run. Subsequently, the eluent composition was changed to the initial starting conditions and the column was reconditioned for the next run for a total of 10 min. With the final conditions, the best possible separation of the included compounds was achieved. However, baseline separation of His, Val-His, and Fru-Val-His, which all co-elute within 15.3 min and 15.8 min, as well as *ipr*Fru and *ipr*Glu, co-eluting within 1.6 min and 1.7 min, was not possible, as shown in Fig. 1. Due to the coupled tandem MS analysis and the accompanying determination of three specific transition ions after CID, all compounds were able to be specifically detected, despite their co-elution. The final retention times (*t*_R_) of the included compounds in the HILIC-ESI-MS/MS method are stated in Table 2. The HILIC method was applied to analyze final fructosamine derivatives, detecting besides the Fru-Val **4a** and Fru-Val-His **4b** small amounts of remaining starting materials Val and Val-His, respectively. Besides the residual amino acid and dipeptide, no further starting material or intermediates were observed, verifying the complete removal of excessive *ipr*Glu by SCX chromatography as well as a complete deprotection of the isopropylidene protecting groups. Since Fru-Val-His **4b** was aimed to be applied in an enzymatic cleavage assay, the amount of Val-His was quantified, recording a calibration curve for Val-His at five different concentrations (see ESM). The amount of Val-His was determined to be 3%, which was taken into account for sample preparations for the subsequent enzymatic cleavage assay of synthetic Fru-Val-His by FPOX.

**Table 2:** Overview of parameters from the HILIC-ESI-MS/MS method, showing most abundant precursor ions, three specific transition ions (most abundant highlighted in italics), optimal collision energies (CE) and cell exit potentials (CXP) for collision-induced dissociation (CID), as well as final retention times (*t*_R_) from HILIC chromatography of all substances included in the HILIC-ESI-MS/MS method. The MS experiments were all performed in positive ESI mode. All precursor ions are monoprotonated adducts if not stated otherwise

Substance	MS/MS parameters					HILIC
	Monoisotopic mass (g/mol)	Precursor ion *m*/*z*	Transition ions *m*/*z*	CE (eV)	CXP (eV)	*t*_R_ (min)
*ipr*Fru **1**	260.1	278.1^a^	*230.1*	*17*	*8*	1.6
			127.0	27	4	
			59.0	47	6	
*ipr*Glu **2**	258.1	294.0^b^	258.8	15	12	1.7
			*200.9*	*19*	*8*	
			142.9	25	6	
Val	117.1	118.0	*72.0*	*17*	*8*	6.8
			57.0	41	6	
			55.0	29	6	
His	155.1	156.0	*110.0*	*21*	*12*	15.3
			93.0	33	12	
			83.0	35	10	
Val-His	254.1	255.0	*156.0*	*23*	*6*	15.5
			110.0	35	12	
			72.1	41	8	
*ipr*Fru-Val **3a**	359.2	360.0	*302.2*	*21*	*14*	2.1
			256.1	31	12	
			84.1	53	10	
*ipr*Fru-Val-His **3b**	496.3	497.2	156.0	41	6	3.3
			*110.0*	*65*	*14*	
			95.0	65	12	
Fru-Val **4a**	279.1	280.1	*262.0*	*19*	*12*	10.0
			244.0	21	12	
			216.0	25	10	
Fru-Val-His **4b**	416.2	417.1	156.1	35	6	15.8
			*110.0*	*53*	*12*	
			95.0	59	12	

^a^*ipr*Fru as [M+NH_4_]^+^, ^b^*ipr*Glu as [M+H_2_O+NH_4_]^+^

### Enzymatic oxidative degradation of fructosamine Fru-Val-His using FPOX

**Scheme 2:**
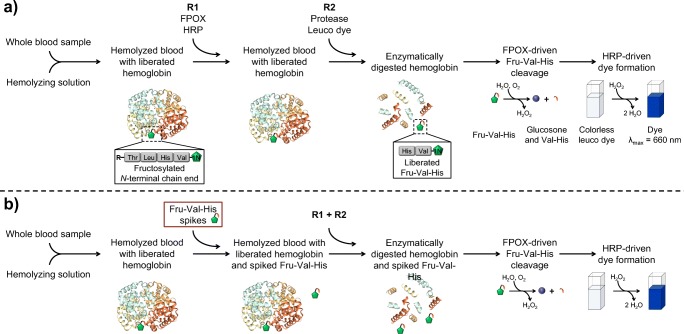
Operating procedure of the enzymatic assay **a** of the commercial assay (DiaSys Diagnostic Systems GmbH) and **b** of the adapted assay with spiked Fru-Val-His **4b**, as performed in this study (both procedures, manually and automatically), see **a** for annotations of structures. Crystal structure of hemoglobin was adapted from RSCB Protein Data Bank [48]. Green pentagon with 1N annotation for fructosamine residue with the amine on carbon atom C1 and blue-white striped sphere for glucosone moiety

To prove the potential of the generated synthetic Fru-Val-His **4b** as a well-defined substrate in the characterization validation of an enzymatic HbA_1c_ assay, enzymatic oxidation was performed using reagent components of a commercially available assay (DiaSys Diagnostic Systems GmbH). During regular HbA_1c_ quantification measurement, a hemolyzed blood sample is incubated for 5 min in the presence of reagent 1 (R1), containing FPOX and horseradish peroxidase (HRP). Since the intact protein HbA_1c_ is not a substrate for the used FPOX, the fructosamine residue does not react with R1. However, during this incubation step, endogenous fructosamine derivatives present in the blood sample are oxidized and the released hydrogen peroxide is degraded by the HRP present, thereby increasing the specificity of HbA_1c_ detection later [47]. After addition of reagent 2 (R2), containing a protease and a leuco dye, HbA_1c_ is enzymatically cleaved by the protease. The liberated Fru-Val-His is subsequently processed by FPOX generating hydrogen peroxide which is used by HRP to generate a dye by oxidizing the leuco dye. The operating procedure of the commercial enzymatic HbA_1c_ assay is shown in Scheme 2a. The measured absorbance of the dye is directly correlated to the Fru-Val-His and thus the HbA_1c_ concentration in the sample.

**Fig. 2:**
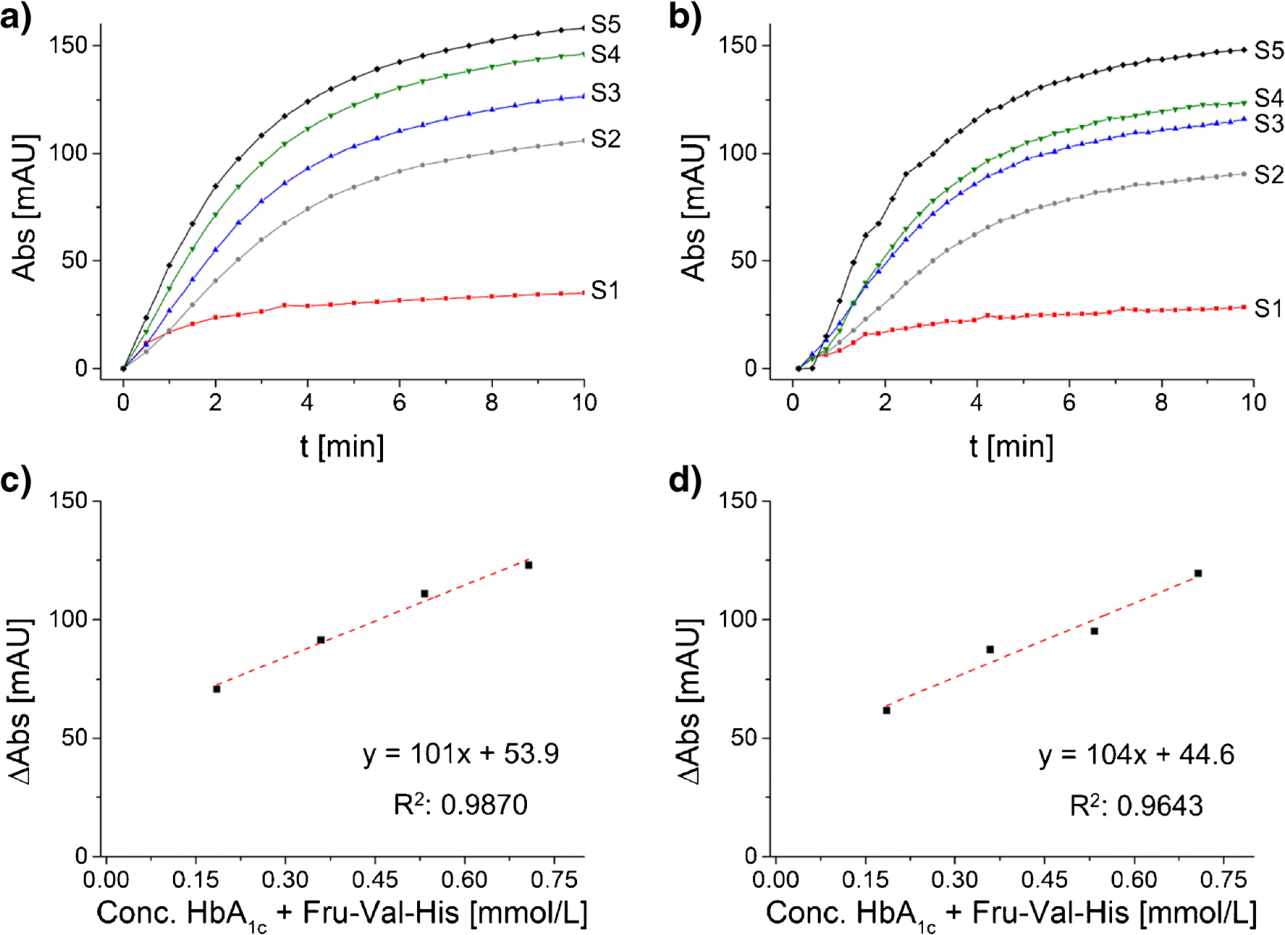
Results from the enzymatic degradation assay of Fru-Val-His **4b** using FPOX showing in **a** the measured absorption increase at 660 nm for the reagent blank (S1), the endogenous HbA_1c_ content of the blood sample (S2), and the three spiking levels (S3–S5) performed manually using the UVIKON XL spectrophotometer and **c** the corresponding linear regression curve for the plot of ΔAbs (ΔAbs of measured samples S2–S5 corrected by blank S1) against the corresponding concentrations of endogenous HbA_1c_ + Fru-Val-His **4b**, taking the dilution of the blood sample by 1/20 into account, with resulting equation and coefficient of determination *R*^2^ for the regression curve. In **b** and **d**, the corresponding data of the automatic measurement by the clinical chemistry analyzer Hitachi 917E are shown

In order to measure purified Fru-Val-His, it is spiked into the hemolysate of a 1/20 dilution of a blood sample with the hemolyzing buffer supplied by the commercial assay (DiaSys Diagnostic Systems). Premixed R1 and R2 components of this assay are subsequently added to the hemolysate, as shown in Scheme 2b. The premixing of R1 and R2 prevents a direct degradation of spiked Fru-Val-His by FPOX in R1 without the presence of the leuco dye and an uncontrolled loss of generated hydrogen peroxide. Depending on the added amount of Fru-Val-His **4b**, a different increase in absorbance could be observed (Fig. 2a). Furthermore, the final absorbance after the 10-min reaction time showed a linear correlation when plotted against the corresponding fructosamine concentrations of 0.185, 0.359, 0.553, and 0.707 mmol/L, taking the endogenous HbA_1c_ value of the blood sample (0.185 mmol/L) into account (Fig. 2c**)**. The chosen Fru-Val-His concentrations in the enzymatic degradation reflect the HbA_1c_ concentration in human blood which varies between about 0.06 and 0.46 mmol/L [3, 49]. Performing the enzymatic assay manually, using an UVIKON XL spectrophotometer from Goebel Instrumentelle Analytik GmbH (Au, Germany), the regression function shows a good coefficient of determination (*R*^2^) of 0.9870 and a slope of 101 mAU/mmol/L Fru-Val-His (Fig. 2a, c). Comparable results were obtained performing the enzymatic assay automatically using a clinical chemistry analyzer Hitachi 917E from Roche Diagnostics (Basel, Switzerland) with a modified assay protocol, achieving an *R*^2^ of 0.9643 and a slope of 104 mAU/mmol/L (Fig. 2b, d), thereby showing good reproducibility among different instruments, considering the non-standard application on the Hitachi instrument. As shown in the activity/time graph (Fig. 2a), synthetic fructosylated Val-His is well recognized and converted by the FPOX enzyme within the usual measuring time of an automated routine clinical chemistry analyzer and a commercial reagent.

## Conclusions

In the presented work, we introduced a synthetic approach towards the two clinically relevant fructosamines Fru-Val and Fru-Val-His via a protected glucosone intermediate. The two synthetic fructosamines were obtained in suitable quantity and purities > 90%. The structures of the synthesized products were fully characterized by different NMR experiments. Besides their synthesis, a routine HILIC-ESI-MS/MS method was developed, including starting materials and intermediates, allowing for high-throughput quality control of synthesized reference fructosamines. Despite their partial co-elution, all compounds were specifically identified by tandem MS operating in MRM mode. Synthetic fructosamine Fru-Val-His was further applied in an established enzymatic oxidative degradation assay using FPOX for HbA_1c_ quantification, proving its functionality as a specific enzymatic substrate. Taken together, we here provide a new, fast, and efficient protocol for the synthesis and purification of well-defined fructosamines in suitable yield and purity that can be used for the validation and standardization of enzymatic assays for HbA_1c_ quantification. Furthermore, the presented synthetic approach can be adapted also for the synthesis of different fructosamines, studying their harmfulness on the organism or the formation of advanced glycation end-products (AGEs). A solid-phase synthesis approach as well as an additional purification of fructosamines by preparative HPLC can be performed if needed in future studies. Synthetic Fru-Val-His material can also be formulated in specific blood-based matrices to be potentially used as an independent control material and evaluated using IFCC-based reference material and quantification methods in a next step. In addition, it could also open new ways to characterize the enzymes processing fructosamines (e.g., fructosamine oxidases) in various applications.

## Electronic supplementary material

ESM 1 (PDF 4234 kb)
