# Supplementary material for: Direct glucosone-based synthesis and HILIC-ESI-MS/MS characterization of N-terminal fructosylated valine and valylhistidine for validation of enzymatic HbA1c assays in the diagnosis of diabetes mellitus
Source: Anal Bioanal Chem. 2019 Nov 22;411(30):7967–79. doi: 10.1007/s00216-019-02186-2 (PMC6920237; doi:10.1007/s00216-019-02186-2)
Supplement: Supplementary file 1 — (PDF 4234 kb) [file 216_2019_2186_MOESM1_ESM.pdf]

Analytical and Bioanalytical Chemistry

Electronic Supplementary Material

Direct glucosone based synthesis and HILIC-ESI-MS/MS characterization of *N*-terminal fructosylated valine and valylhistidine for validation of enzymatic HbA_{1c} assays in the diagnosis of *Diabetes mellitus*

Christoph Gerke, Monika Buchholz, Holger Müller, Reinhard Meusinger, Matthias Grimmler, Erwin Metzmann

Instrumentation

Nuclear Magnetic Resonance spectroscopy (NMR)

NMR spectra were recorded on a Bruker DRX500 (^{1}H NMR 500 MHz and ^{13}C 126 MHz) instrument. Chemical shifts of all NMR spectra were reported in delta (δ) expressed in parts per million (ppm). For structure *ipr*Fru-Val **3a**, residual trimethylsilane (TMS) was used as internal standard to calibrate the chemical shift for ^{1}H and ^{13}C NMR (both to δ 0 ppm). For the other compounds, the signal of residual solvent was used as internal standard. For ^{1}H NMR δ 2.50 ppm for DMSO-d_6 and 7.26 ppm for $CDCl_3$ as well as for ^{13}C NMR δ 39.52 ppm for DMSO-d_6 and 77.16 ppm for $CDCl_3$. The following abbreviations are used to indicate the multiplicities: s, singlet; d, doublet; dd, doublet of doublets; dp, doublet of quartets; ddd, doublet of doublets of doublets; t, triplet; q, quartet; m multiplet.

Furthermore, ^{1}H-^{1}H-COSY, ^{1}H-^{13}C-HSQC and ^{1}H-^{13}C-HMBC measurements were performed. All measurements were performed at 25 °C if not stated otherwise.

Hydrophilic Interaction Liquid Chromatography – Electrospray Ionization – Tandem Mass Spectrometry (HILIC-ESI-MS/MS)

As software for data analysis Analyst ® 1.5.1 software from AB SCIEX (Framingham, USA) was used. The ESI source was operated in positive ionization mode in an *m/z* range of 100 to 700. The temperature of the column compartment was set to 25 °C.

For HILIC method development, a mix-standard with all compound included in the method was prepared. Concentrations of compounds in the mix-standard used for HILIC method development are listed in **Table S1**.

Table S1 Included compounds in the HILIC method and their concentrations in the mix-standard used for method development

Included substance	Concentration [µg/mL]
Val	7.00
His	46.3
Val-His	12.5
*ipr*Fru **1**	67.5
*ipr*Glu **2**	95.0
*ipr*Fru-Val **3a**	6.5
*ipr*Fru-Val-His **3b**	11.3
Fru-Val **4a**	56.3
Fru-Val-His **4b**	138

High Resolution - Mass Spectrometry (HRMS)

HRMS measurements were performed HRMS measurements were performed with an Orbitrap Velos Pro system from Thermo Fisher Scientific Inc (Waltham, USA), with a direct inlet via syringe pump and an Ion Max Housing with ESI source. Analysis by HRMS was done via a syringe pump at a flow rate of 3 µL/min. From each sample, a full scan spectrum in an *m/z* range from 100 till 700 and a resolution of 100

000 was recorded in positive mode. Sum formulas were deduced using defined search criteria to narrow down the number of suggested sum formulas. As search criteria, appropriate values were chosen that fit to the synthesized substances. The entered search criteria for the sum formula generation are listed in **Table S2**.

Table S2 Search criteria for the sum formula deduction in HRMS

Element	Minimum Amount	Maximum Amount
^{1}H	5	40
^{12}C	5	25
^{14}N	0	4
^{16}O	2	8
^{23}Na	0	1

Direct Electrospray Ionization – Mass Spectrometry (Direct ESI-MS)

For direct ESI-MS measurements via syringe pump an ion trap LCQ Mass Spectrometer System from Finnigan MAT (San José, USA) as well as the triple quadrupole instrument LC/MS/MS system API 2000 with electrospray ionization (ESI) source from AB SCIEX (Framingham, USA) were used. For a fast verification of a synthesized substance, solutions of approximately 20-70 µg/mL in a mixture of H_2O and ACN in a ratio of 70/30 (v/v), containing an additional 5 mM NH_4HCO_2 were prepared. The samples were injected into the MS system via syringe pump operating at 10 µL/min and analyzed in scan mode detecting the *m/z* of the isolated substance. Each sample was analyzed in full scan in a set mass range from *m/z* of 100 to 700. Positive as well as negative mode was used. Furthermore, fragmentation of a detected substance was performed for its confirmation (using the LC/MS/MS instrument from AB SCIEX only).

Fourier-Transform Infrared spectroscopy (FTIR)

For IR analysis an FT-IR system Spectrum BX and IR Accessory Hydraulic Press from Perkin Elmer Inc. (Waltham, USA) were used. Crystalline products were analyzed as solid potassium bromide (KBr) disk. Oily products were analyzed by applying on plates of sodium chloride (NaCl) as a thin film.

Melting Point analysis (M. p.)

The melting points were determined with a Stuart melting point determination instrument SMP10 from Bibby Scientific Ltd. (Stone, UK).

Analytical data

2,3:4,5 di-*O*-isopropylidene-β-D-fructopyranose (*iprFru*, 1)

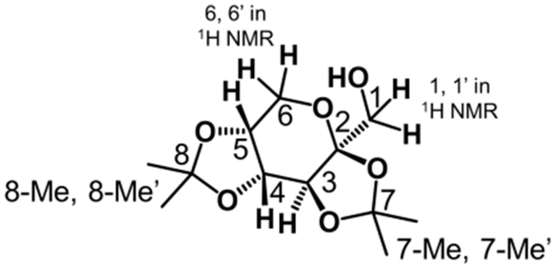

Table S3 Assigned signals from ^{1}H and ^{13}C NMR spectra of *iprFru* **1**, showing chemical shifts for both spectra as well as proton amounts, multiplicity and coupling constants for ^{1}H NMR

**No.**	**^{1}H NMR**				**^{13}C NMR**
	**Shift**	**Amount**	**Multiplicity**	**Coupling constant**	**Shift**
1	3.64	1	dd	11.7, 4,4	65.79
1'	3.69	1	dd	11.7, 8.4	
2	-	-	-	-	103.26
3	4.32	1	dd	2.6	71.24
4	4.59	1	dd	7.9, 2.6	70.26
5	4.22	1	ddd	7.9, 2.0, 0.7	70.99
6	3.76	1	dd	13.0, 0.5	61.49
6'	3.90	1	dd	13.0, 2.0	
7	-	-	-	-	108.71 [a)]
8	-	-	-	-	109.27 [a)]
7-Me, 7-Me'	1.38, 1.53 [a)]	3, 3	s, s	-	25.53, 26.65 [a)]
8-Me, 8Me'	1.33, 1.46 [a)]	3, 3	s, s	-	24.17, 25.96 [a)]
-OH	2.06	1	dd	8.5, 4.7	-

[a)] interchangeable

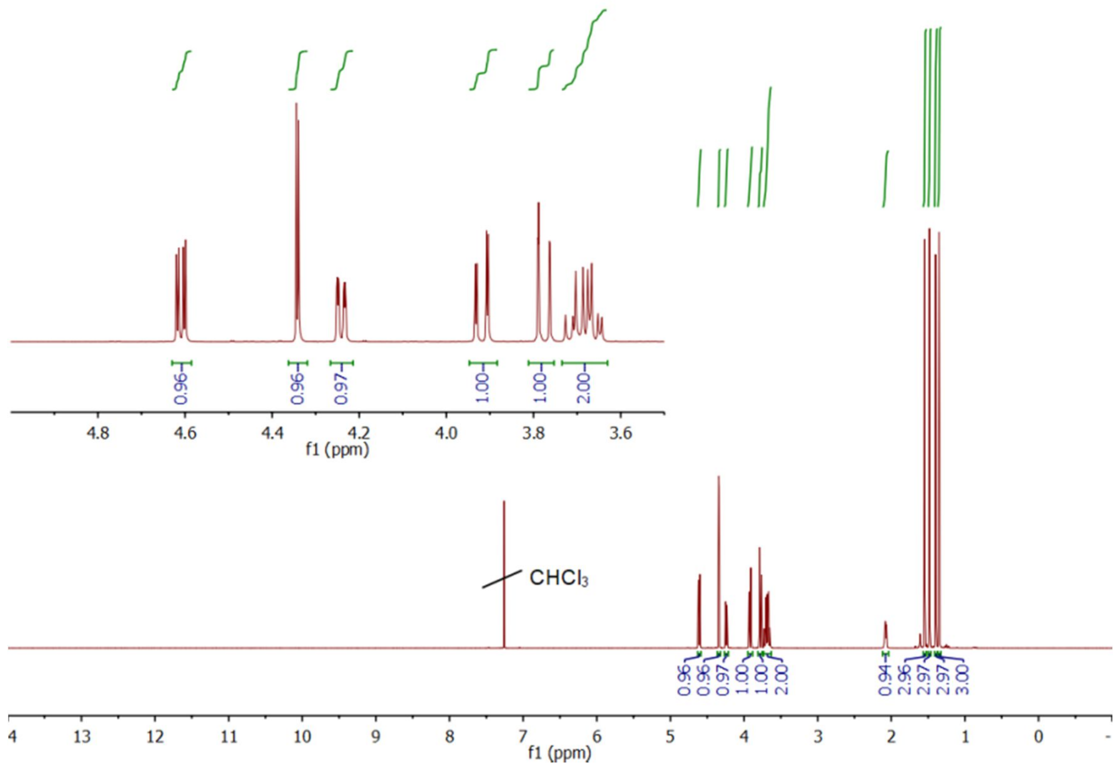

Fig. S1 ^{1}H NMR (500 MHz, $CDCl_3$) spectrum of *ipr*Fru **1** with the enlarged area from δ 5.0 to 3.5 ppm

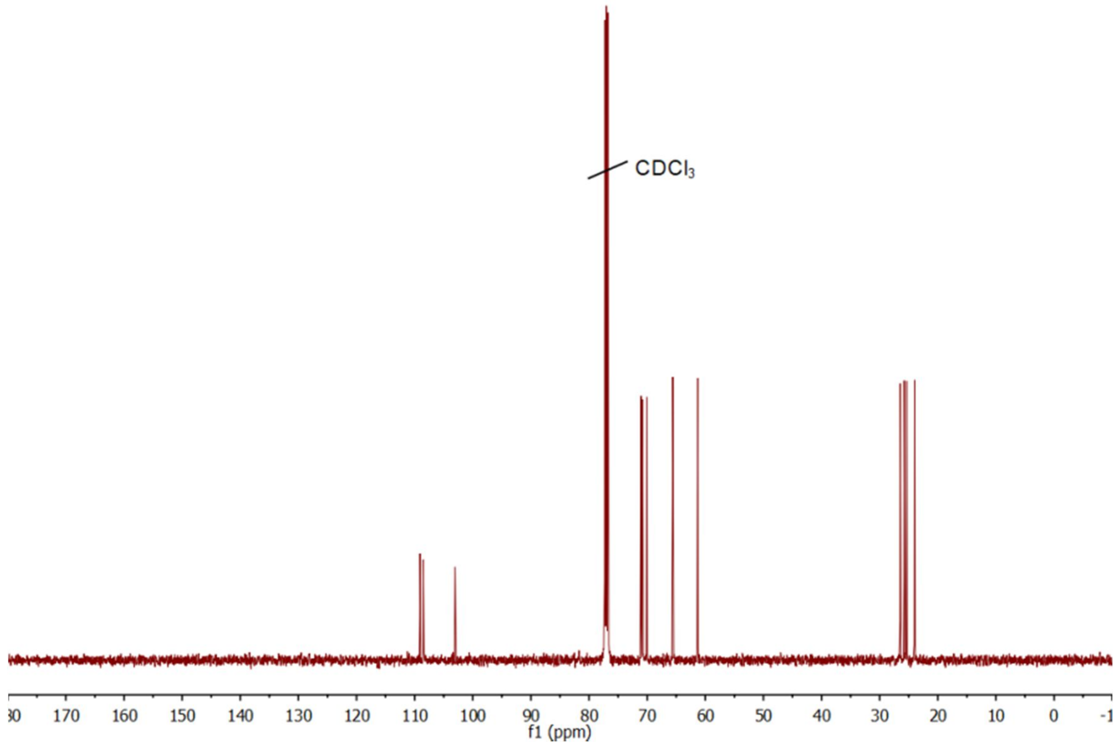

Fig. S2 ^{13}C NMR (126 MHz, $CDCl_3$) spectrum of *ipr*Fru **1**

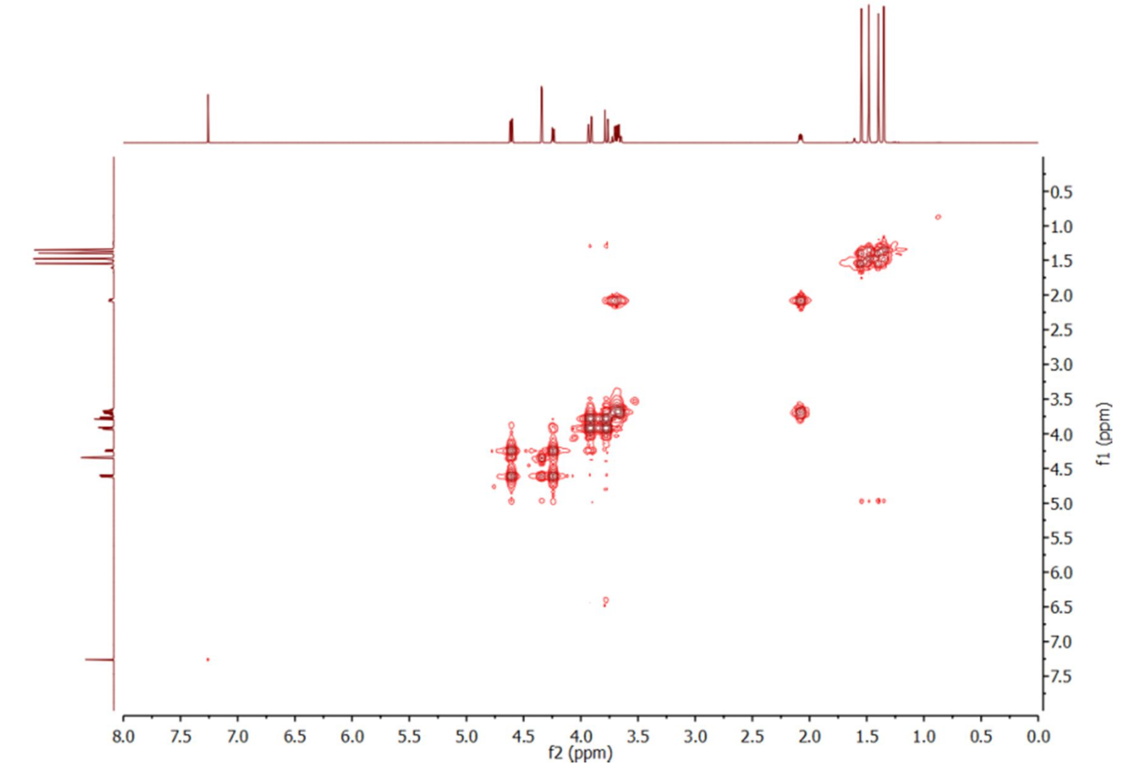

Fig. S3 ^{1}H-^{1}H-COSY spectrum of *ipr*Fru **1**

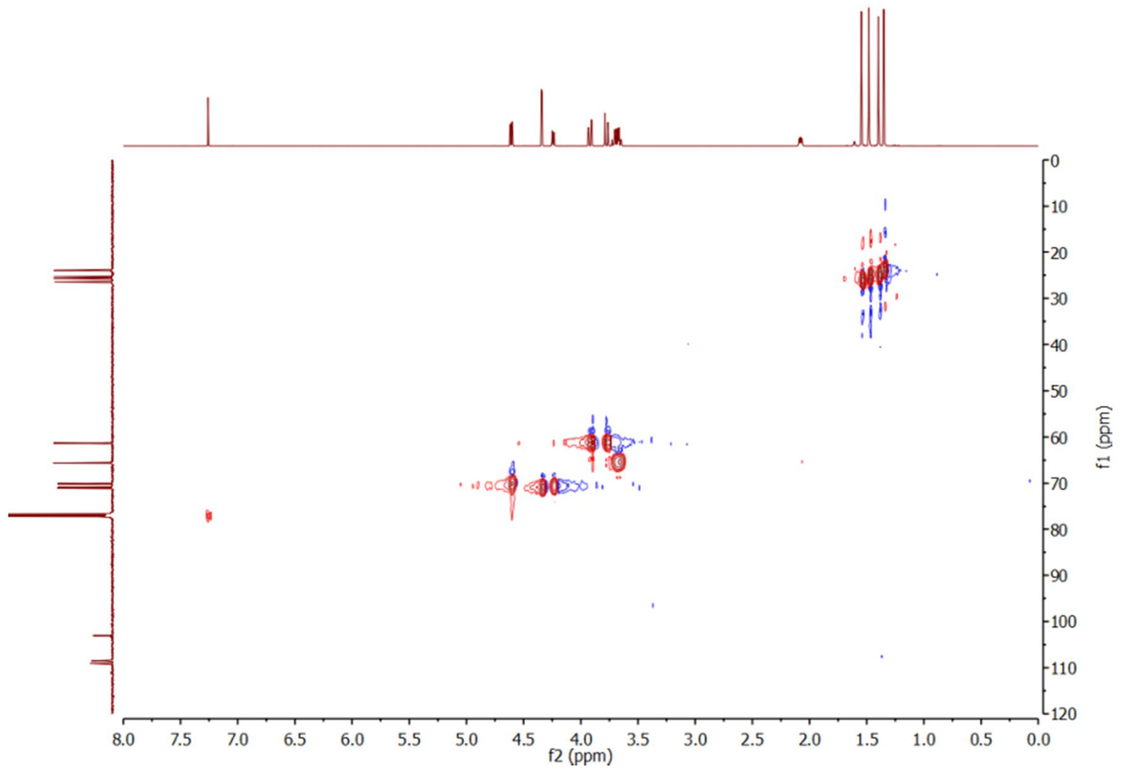

Fig. S4 ^{1}H-^{13}C-HSQC spectrum of *ipr*Fru **1**

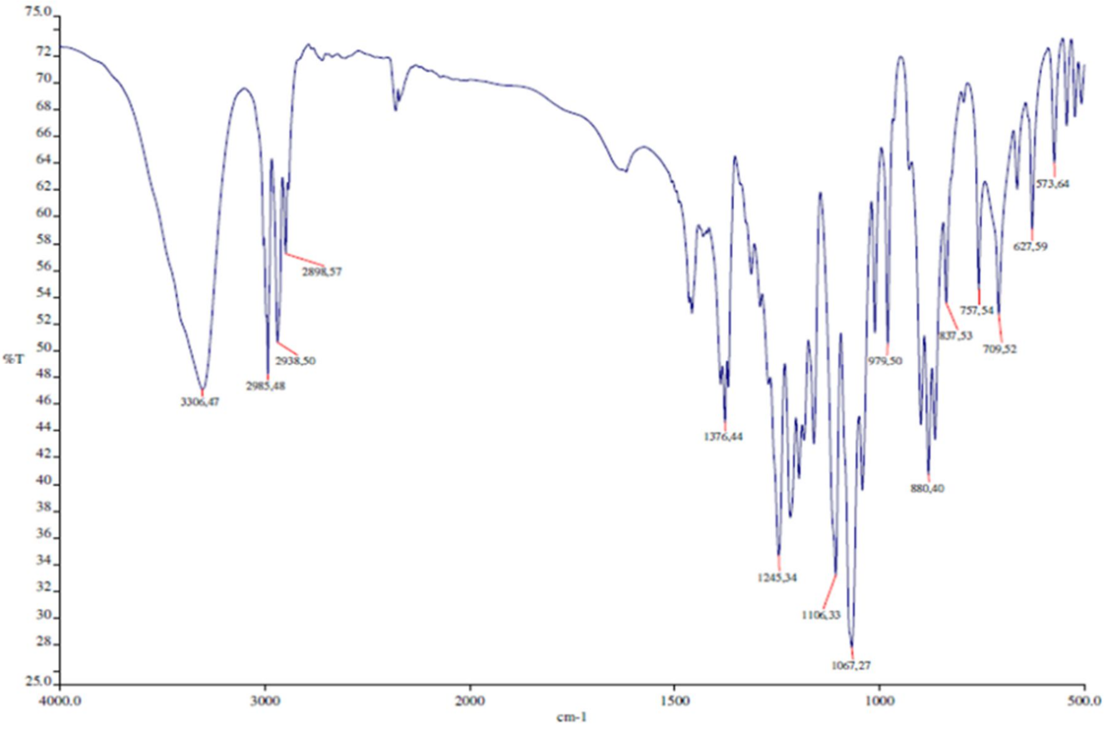

Fig. S5 FT-IR (KBr disk) spectrum of *ipr*Fru **1**

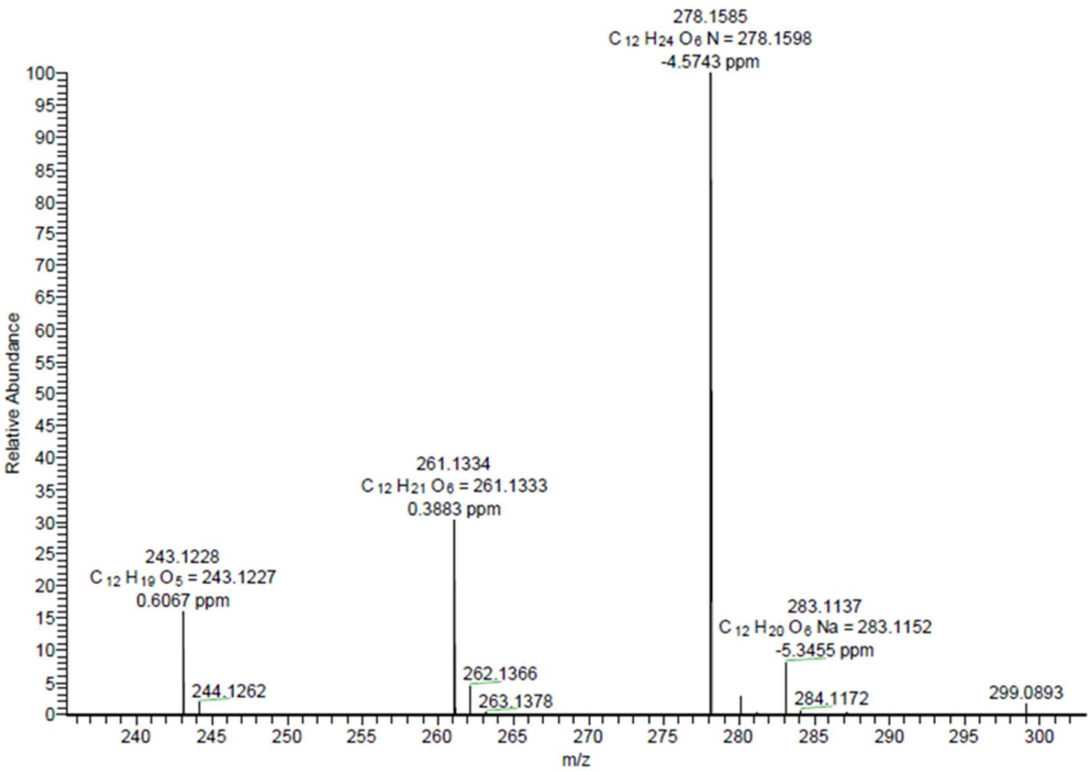

Fig. S6 HRMS (ESI$^+$ Orbitrap) spectrum of *ipr*Fru **1**

2,3:4,5-di-O-isopropylidene-aldehydo-β-D-arabinohexos-2-ulo-2,6-pyranose (*ipr*Glu, 2) and its hydrate form

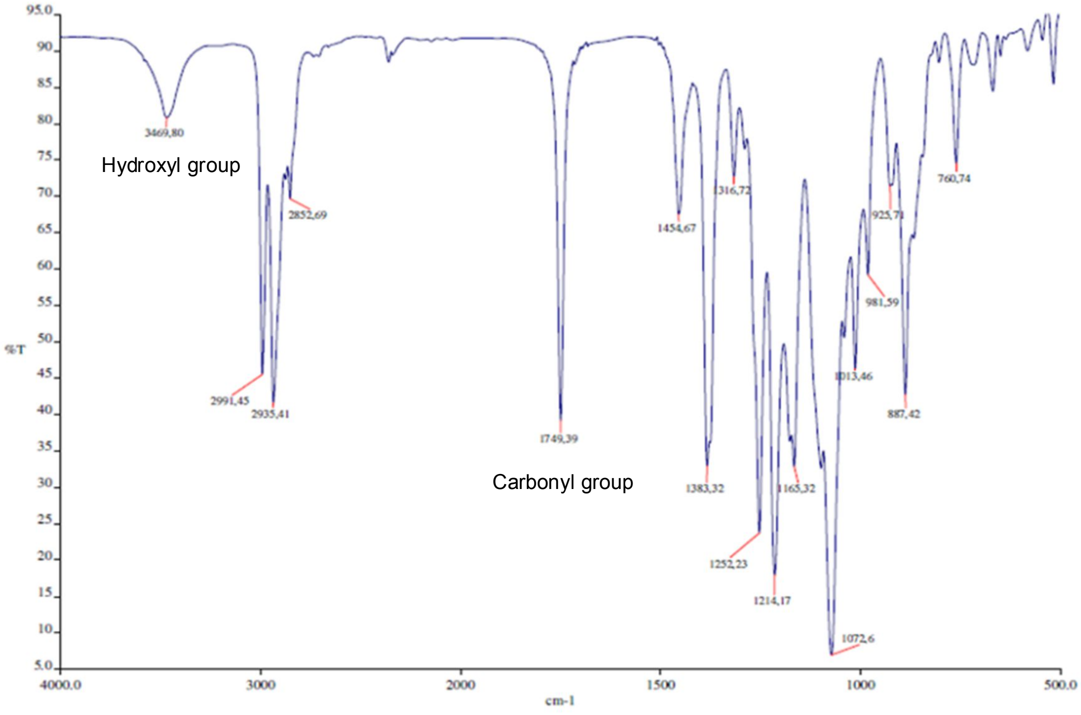

Fig. S7 FT-IR (film on NaCl) spectrum of *ipr*Glu **2**

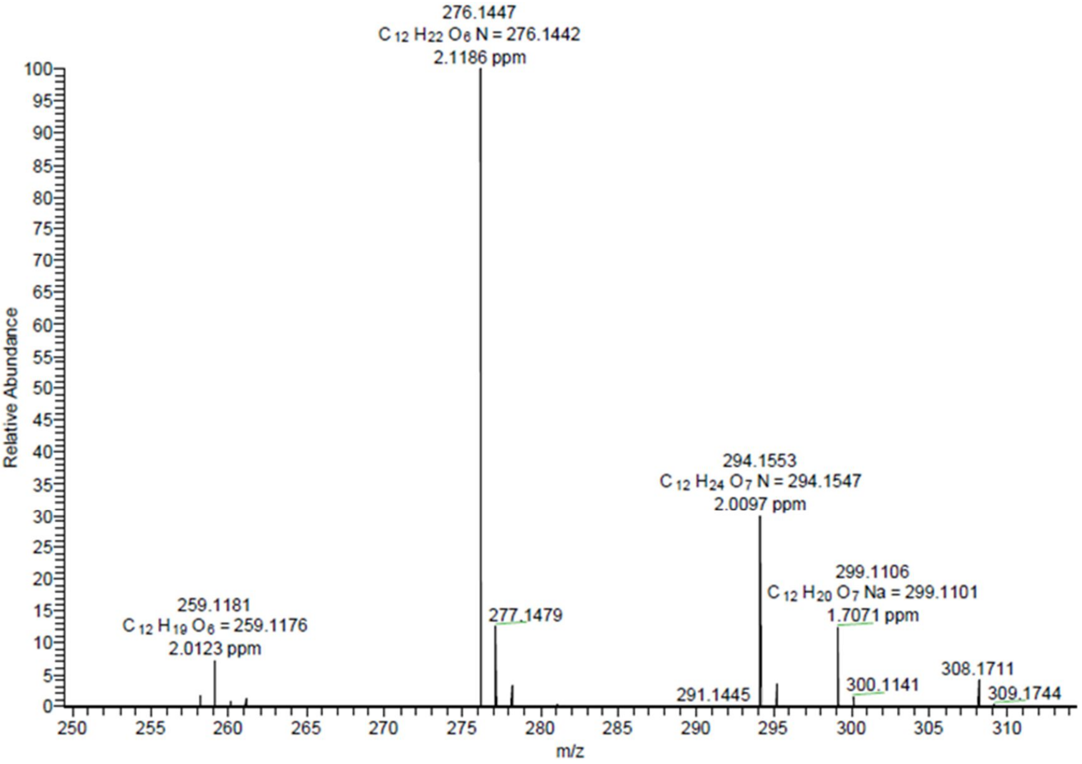

Fig. S8 HRMS (ESI+ Orbitrap) spectrum of *ipr*Glu **2**

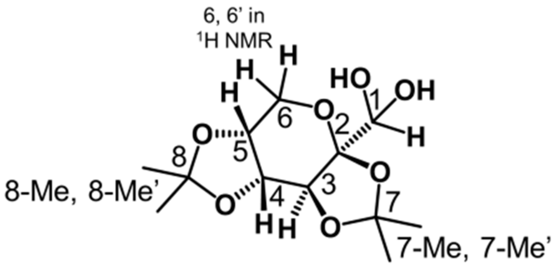

Table S4 Assigned signals from ^{1}H and ^{13}C NMR spectra of *ipr*Glu **2**, showing chemical shifts for both spectra as well as proton amounts, multiplicity and coupling constants for ^{1}H NMR

No.	^{1}H NMR				^{13}C NMR
	Shift	Amount	Multiplicity	Coupling constant	Shift
1	4.68	1	t	6.6	89.32
2	-	-	-	-	103.52
3	4.33	1	d	2.8	69.84
4	4.54	1	dd	8.0, 2.6	69.49
5	4.20	1	dd	8.0, 1.5	70.14
6	3.57	1	d	13.0	60.15
6'	3.72	1	dd	13.1, 1.9	
7	-	-	-	-	108.01 [a)]
8	-	-	-	-	107.82 [a)]
7-Me, 7-Me'	1.27, 1.36 [a)]	3, 3	s, s	-	25.53, 26.47 [a)]
8-Me, 8Me'	1.36, 1.44 [a)]	3, 3	s, s	-	23.99, 25.57 [a)]
-OH	5.69, 5.62	1, 1	d, d	6.8, 6.4	-

[a)] interchangeable

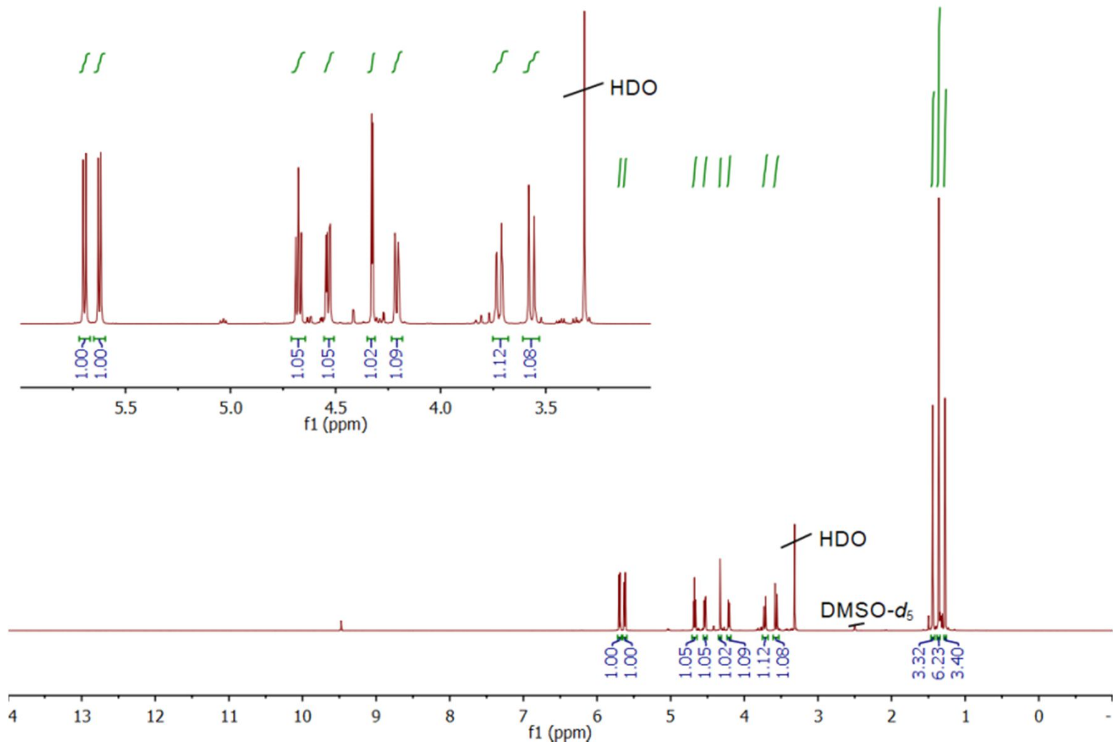

Fig. S9 ^{1}H NMR (500 MHz, DMSO-d_6) spectrum of *ipr*Glu **2** with the enlarged area from δ 6.0 to 3.0 ppm

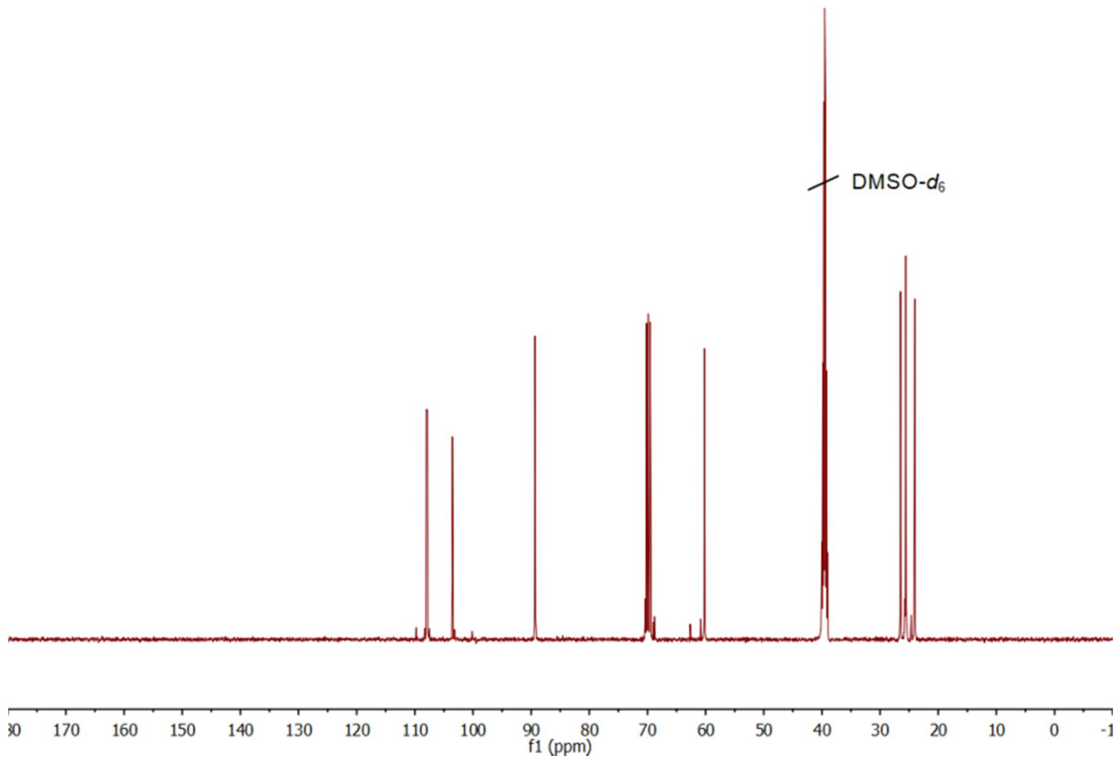

Fig. S10 ^{13}C NMR (126 MHz, DMSO-d_6) spectrum of *ipr*Glu **2**

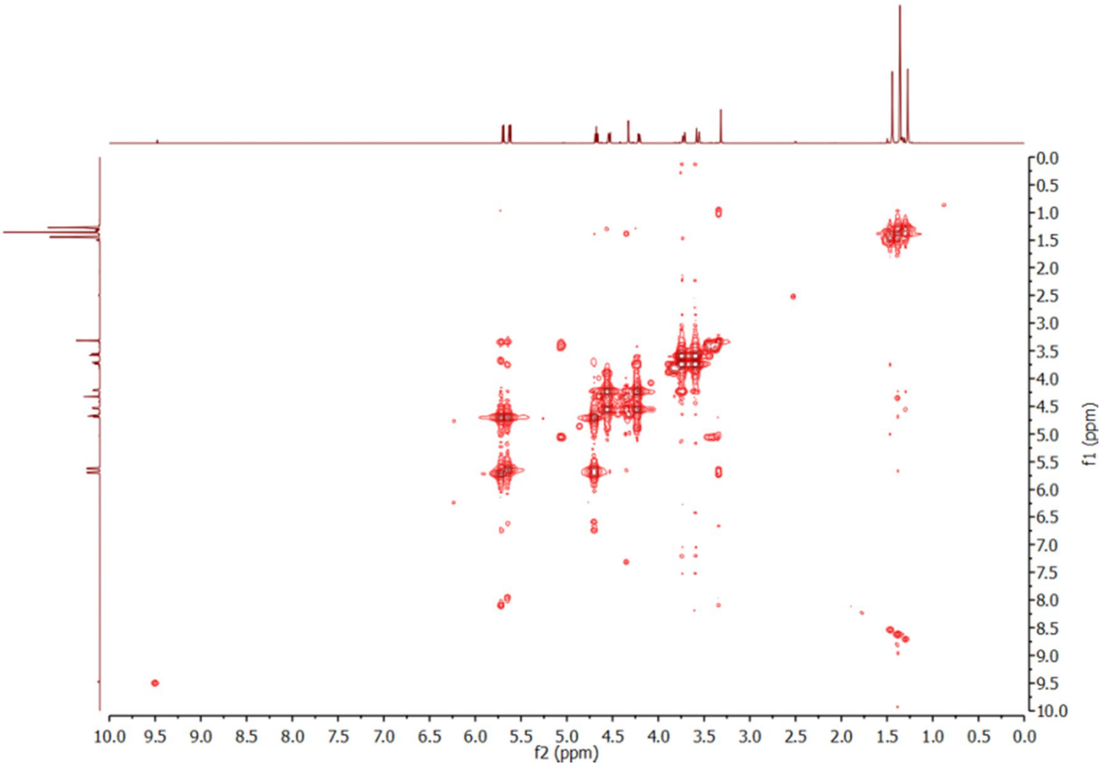

Fig. S11 ^{1}H-^{1}H-COSY spectrum of *ipr*Glu **2**

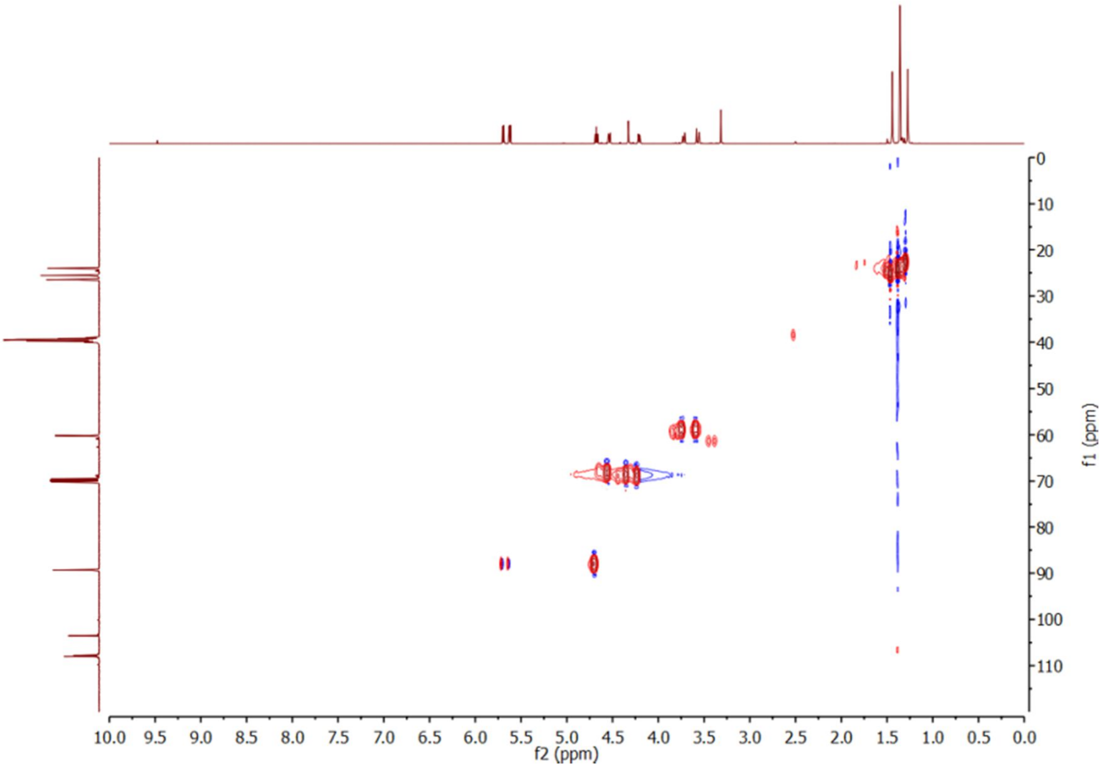

Fig. S12 ^{1}H-^{13}C-HSQC spectrum of *ipr*Glu **2**

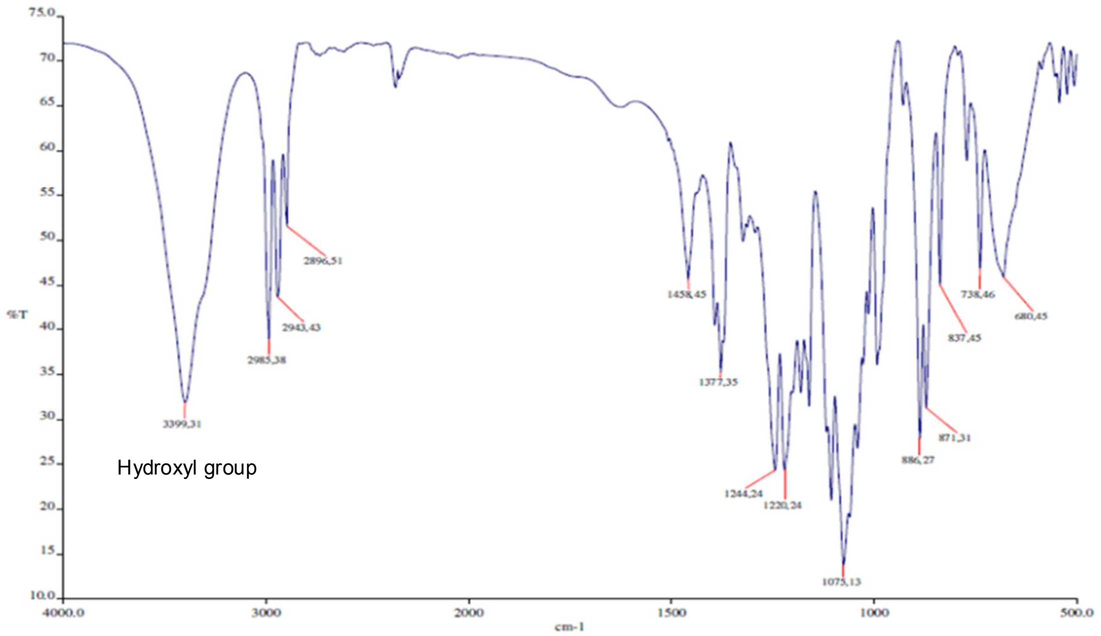

Fig. S13 FT-IR (KBr disk) spectrum of *ipr*Glu **2** – hydrate

Protected fructosylated Val (*ipr*Fru-Val, 3a)

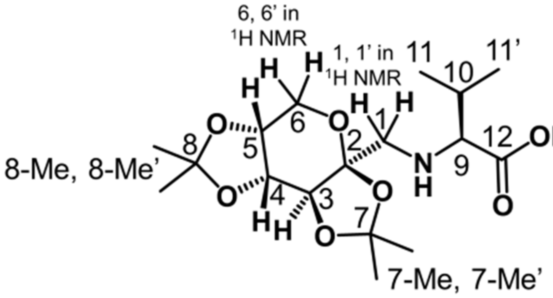

Table S5 Assigned signals from ^{1}H and ^{13}C NMR spectra of *ipr*Fru-Val **3a**, showing chemical shifts for both spectra as well as proton amounts, multiplicity and coupling constants for ^{1}H NMR

No.	^{1}H NMR				^{13}C NMR
	Shift	Amount	Multiplicity	Coupling constant	Shift
1	2.64	1	d	12.0	57.27
1'	3.05	1	d	12.0	
2	-	-	-	-	102.48
3	4.02	1	d	2.6	72.49
4	4.52	1	dd	7.9, 2.5	70.29
5	4.17	1	dd	7.9, 1.8	70.95
6	3.70	1	d	13.0	61.49
6'	3.81	1	dd	13.0, 2.0	
7	-	-	-	-	109.31 [a)]
8	-	-	-	-	108.79 [a)]
7-Me, 7-Me'	1.30, 1.39 [a)]	3, 3	s, s	-	25.19, 26.10 [a)]
8-Me, 8Me'	1.28, 1.48 [a)]	3, 3	s, s	-	24.23, 26.54 [a)]
9	3.03	1	d	4.0	67.70
10	2.25 – 2.14	1	m	-	31.06
11	0.90	3	d	6.9	17.92
11'	0.97	3	d	7.0	19.38
12	-	-	-	-	174.11

[a)] interchangeable

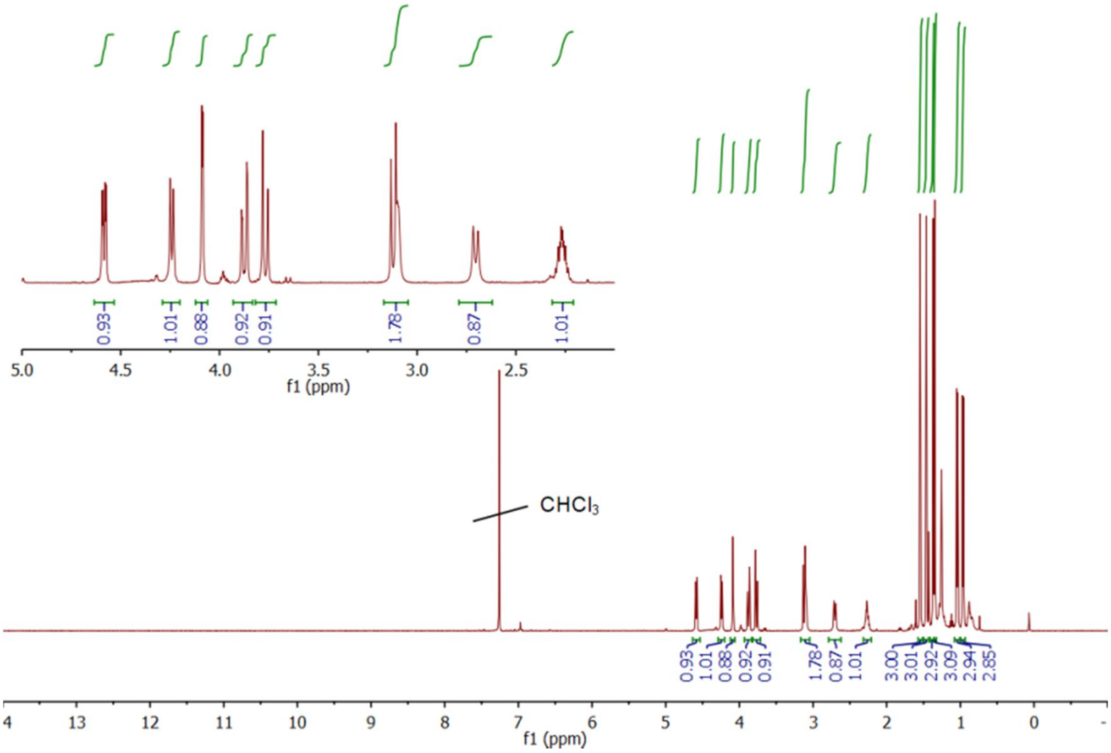

Fig. S14 ^{1}H NMR (500 MHz, CDCl$_3$) spectrum of *ipr*Fru-Val **3a** with the enlarged area from δ 5.0 to 2.0 ppm

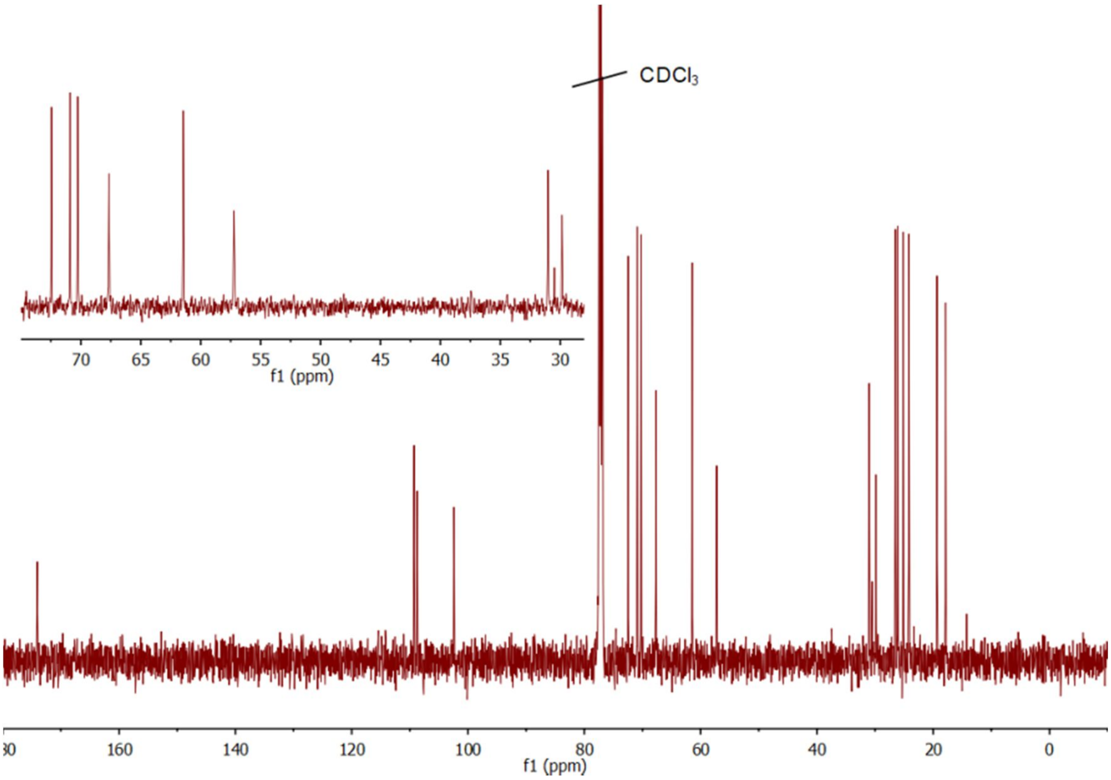

Fig. S15 ^{13}C NMR (126 MHz, CDCl$_3$) spectrum of *ipr*Fru-Val **3a** with the enlarged area from δ 78 to 28 ppm

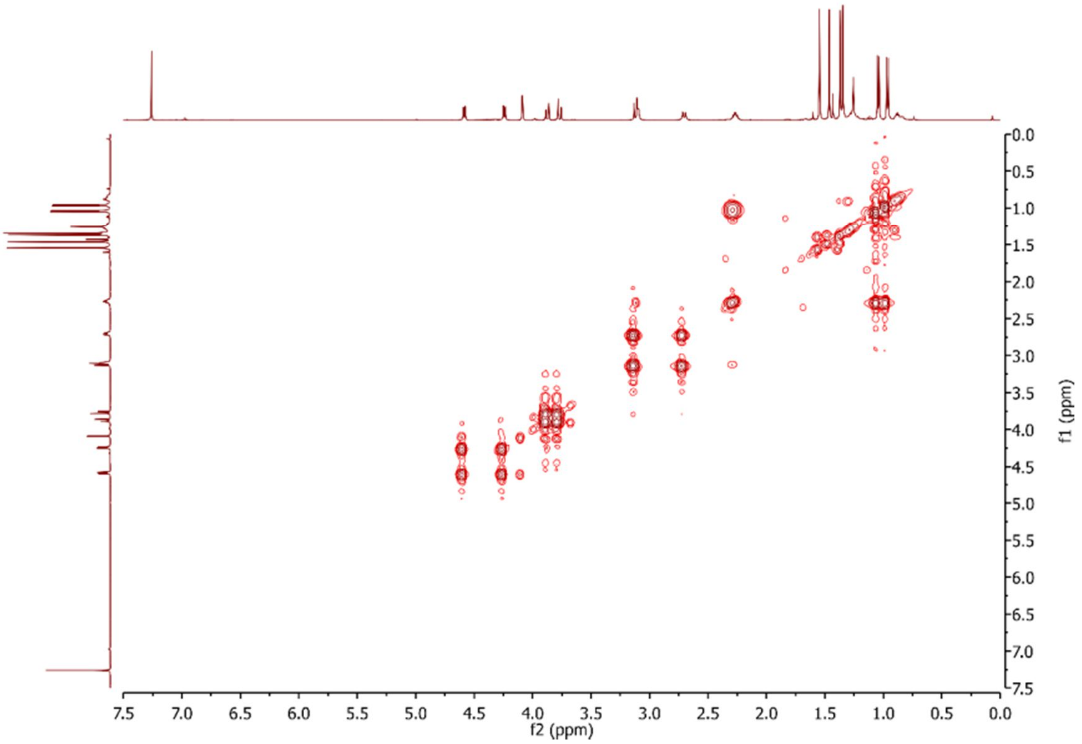

Fig. S16 ^{1}H-^{1}H-COSY spectrum of *ipr*Fru-Val **3a**

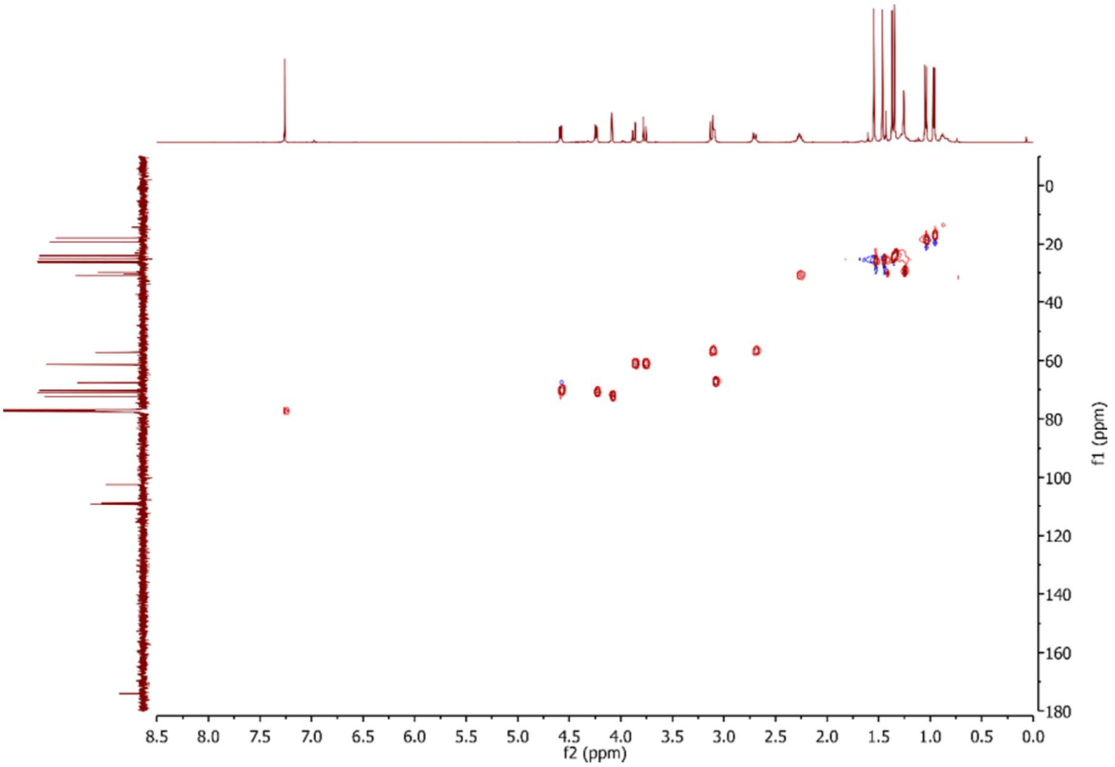

Fig. S17 ^{1}H-^{13}C-HSQC spectrum of *ipr*Fru-Val **3a**

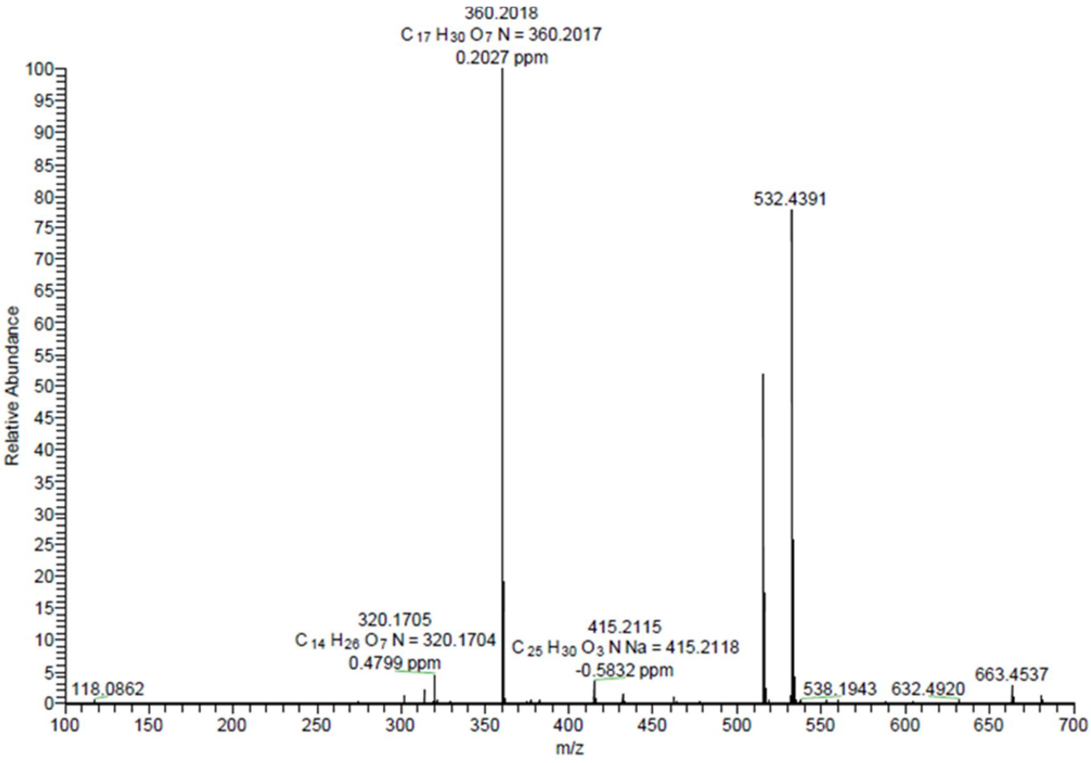

Fig. S18 HRMS (ESI+ Orbitrap) spectrum of *ipr*Flu-Val **3a**

Protected fructosylated Val-His (*ipr*Fru-Val-His, 3b)

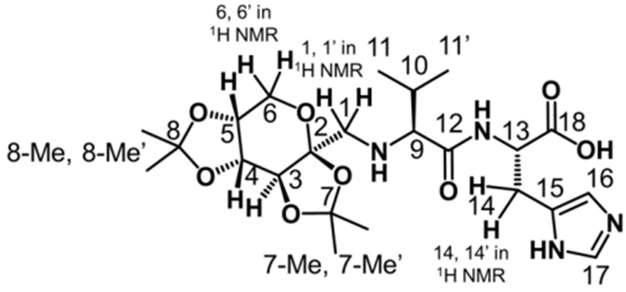

Table S6 Assigned signals from ^{1}H and ^{13}C NMR spectra of *ipr*Fru-Val-His **3b**, showing chemical shifts for both spectra as well as proton amounts, multiplicity and coupling constants for ^{1}H NMR

No.	^{1}H NMR				^{13}C NMR
	Shift	Amount	Multiplicity	Coupling constant	Shift
1	2.60	1	d	12.3	53.97
1'	2.74	1	d	12.4	
2	-	-	-	-	103.00
3	4.36	1	d	2.6	70.77
4	4.52	1	dd	7.9, 2.5	69.66
5	4.19	1	d	7.8	70.16
6	3.54	1	d	13.0	60.36
6'	3.73	1	d	12.9	
7	-	-	-	-	107.29 [a)]
8	-	-	-	-	107.93 [a)]
7-Me, 7-Me'	1.34, 1.44 [a)]	3, 3	s, s	-	25.30, 26.28 [a)]
8-Me, 8Me'	1.26, 1.33 [a)]	3, 3	s, s	-	23.97, 25.70 [a)]

No.	^{1}H NMR				^{13}C NMR
	Shift	Amount	Multiplicity	Coupling constant	Shift
9	2.78	1	d	5.7	67.82
10	1.82	1	dp	13.3, 6.7	30.74
11	0.80	3	d	6.8	18.48
11’	0.80	3	d	6.8	18.99
12	-	-	-	-	172.93
13	4.28	1	q	6.6	52.36
14	2.88	1	dd	14.7, 7.0	29.00
14’	2.95	1	dd	14.8, 5.6	
15	.	-	-	-	132.61
16	6.75	1	s	-	118.00
17	7.48	1	s	-	134.38
18	.	-	-	-	172.31
NH_2 (between 12 and 13)	7.94	1	d	7.1	-

a) interchangeable

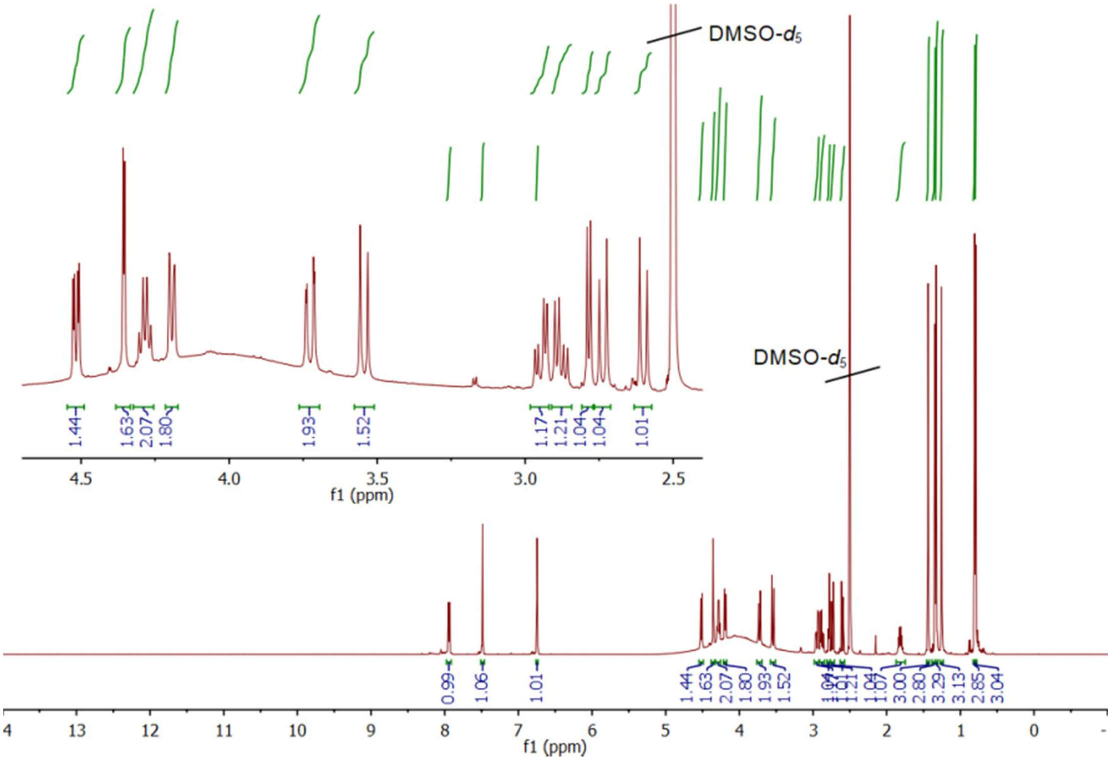

Fig. S19 ^{1}H NMR (500 MHz, DMSO-d_6) spectrum of *ipr*Fru-Val-His **3b** with the enlarged area from δ 4.6 to 2.4 ppm

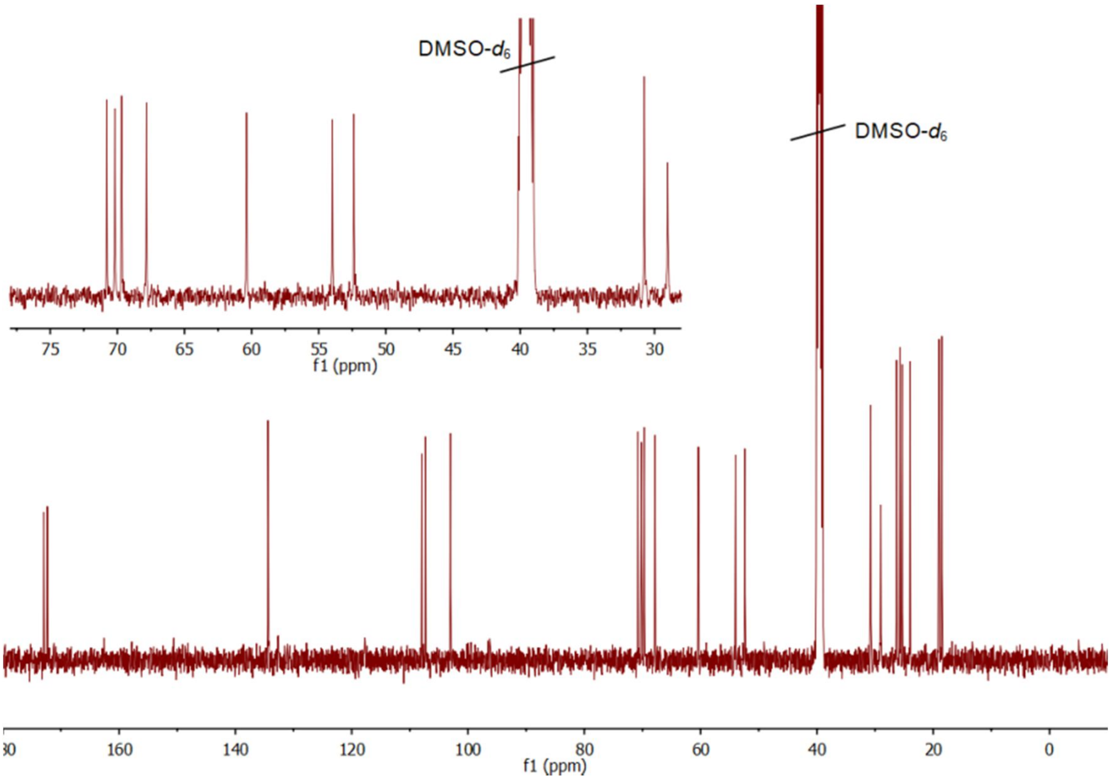

Fig. S20 ^{13}C NMR (126 MHz, DMSO-d_6) spectrum of *ipr*Fru-Val-His **3b** with the enlarged area from δ 78 to 28 ppm

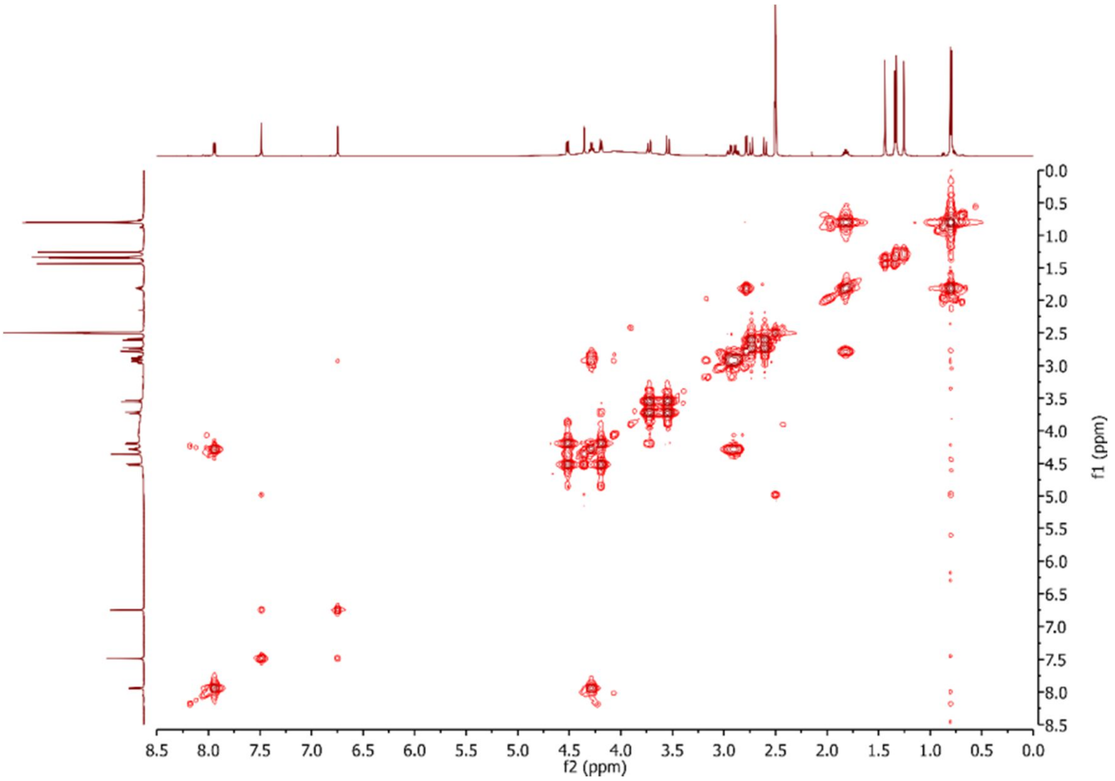

Fig. S21 ^{1}H-^{1}H-COSY spectrum of *ipr*Fru-Val-His **3b**

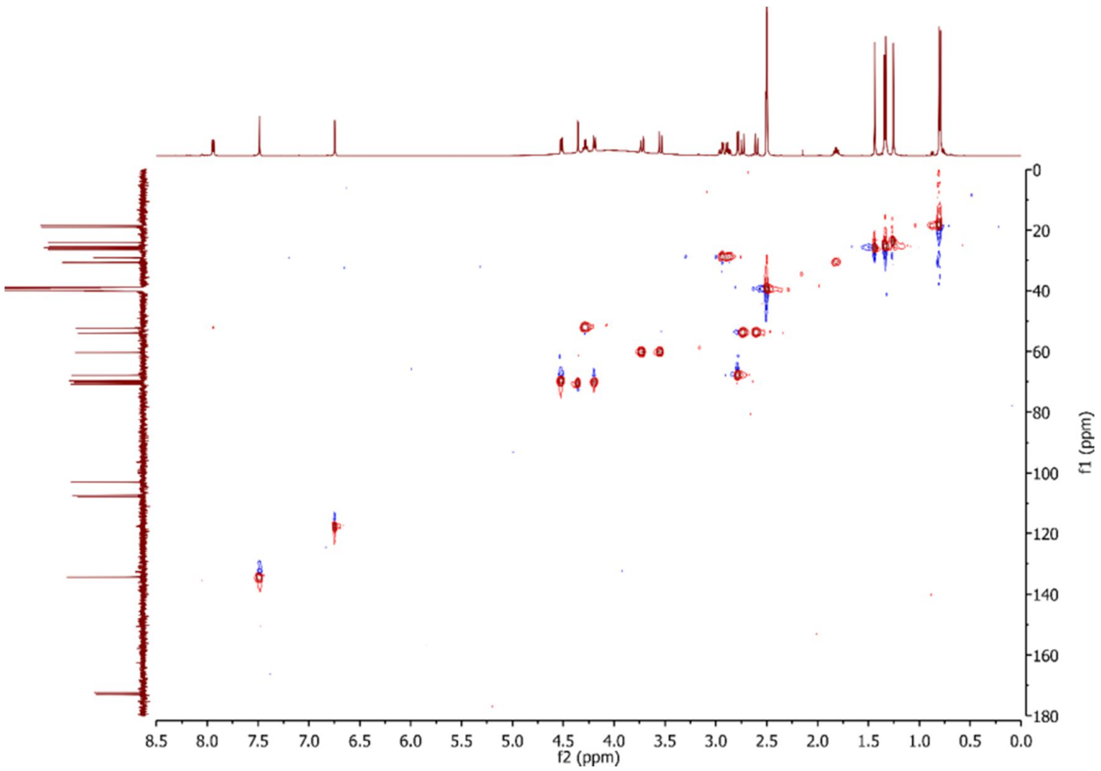

Fig. S22 ^{1}H-^{13}C-HSQC spectrum of *ipr*Fru-Val-His **3b**

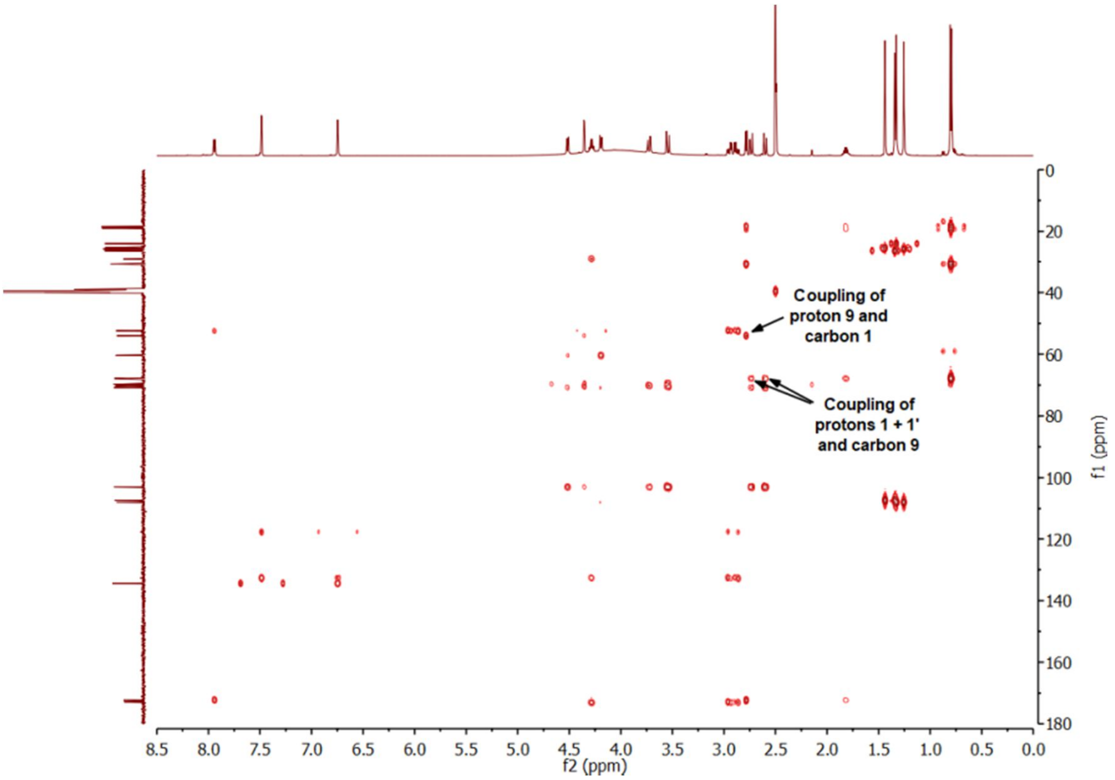

Fig. S23 ^{1}H-^{13}C-HMBC spectrum of *ipr*Fru-Val-His **3b**, with the highlighted coupling signals of proton 9 and carbon 1 as well as protons 1 + 1' and the carbon 9, thereby proving the correct linkage of the fructosyl moiety to the dipeptide

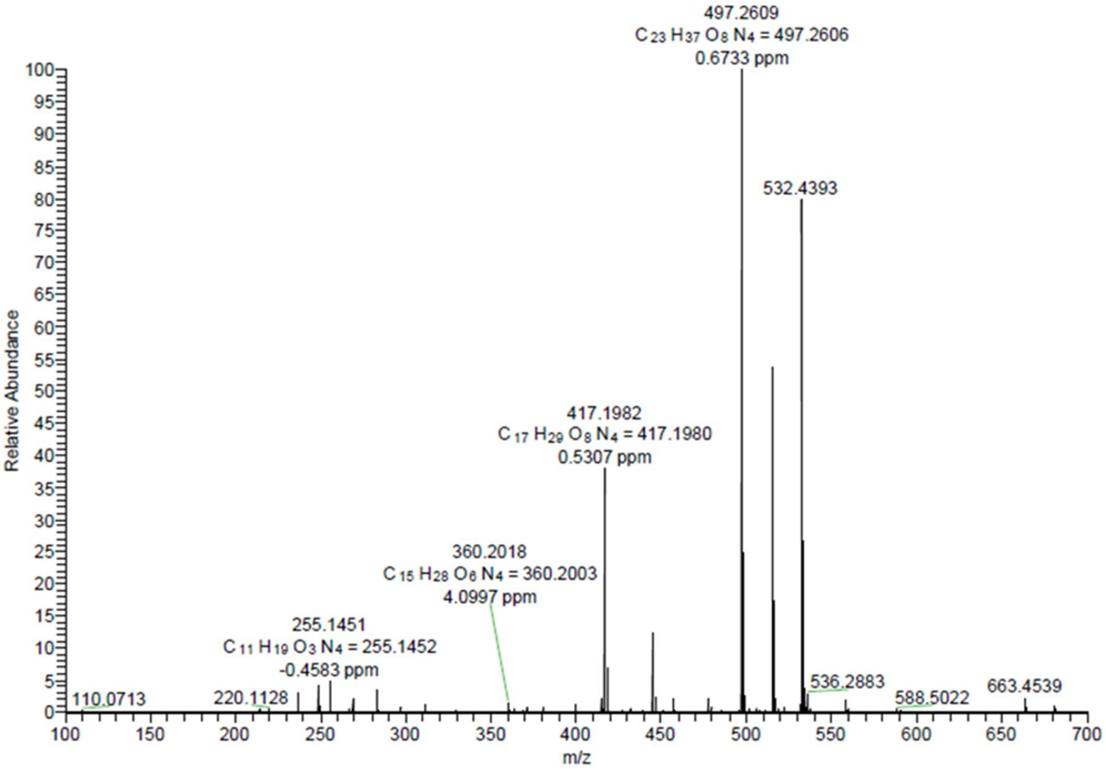

Fig. S24 HRMS (ESI+ Orbitrap) spectrum of *ipr*Flu-Val-His **3b**

Fructosylated Val (Fru-Val, 4a)

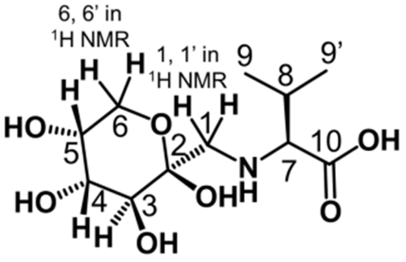

Table S7 Assigned signals from ^{1}H spectrum of Fru-Val **4b**, showing chemical shifts, proton amounts, multiplicity and coupling constants for ^{1}H NMR

No.	^{1}H NMR			
	**Shift**	**Amount**	**Multiplicity**	**Coupling constant**
1<br>1'	3.02 – 2.63	2	m	-
2	-	-	-	-
3<br>4<br>5<br>6<br>6'	3.93 – 3.46	5	m	-
7	3.20	1	broad	-
8	2.30 – 1.86	1	m	-
9<br>9'	0.99 – 0.82	6	m	-
10	-	-	-	-

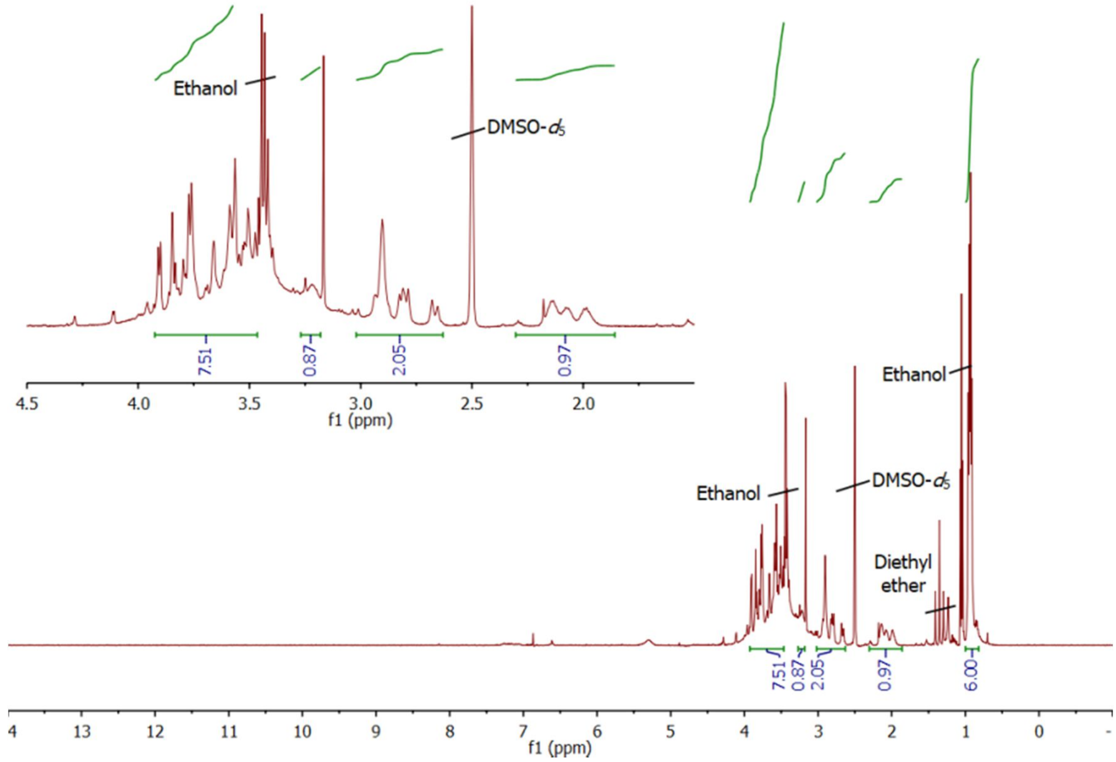

Fig. S25 ^1H NMR (500 MHz, DMSO-d_6) spectrum of Fru-Val **4a** with the enlarged area from δ 4.5 to 1.5 ppm

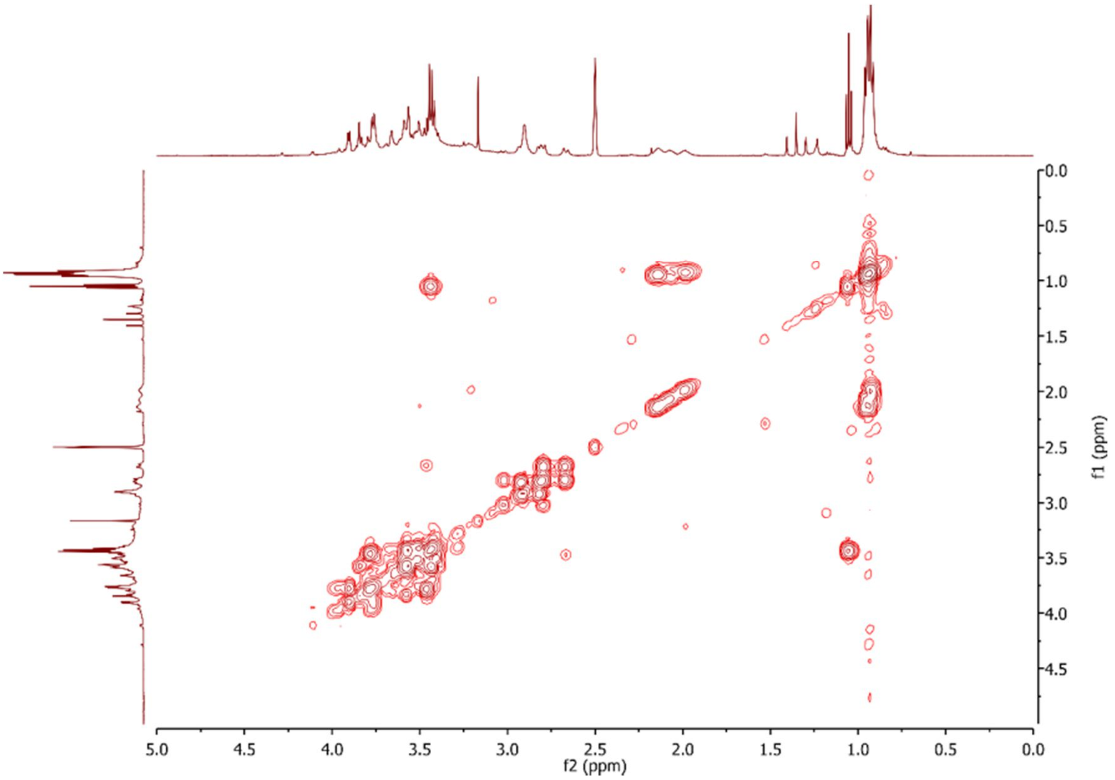

Fig. S26 ^1H-^1H-COSY spectrum of *ipr*Fru-Val-His **4a**

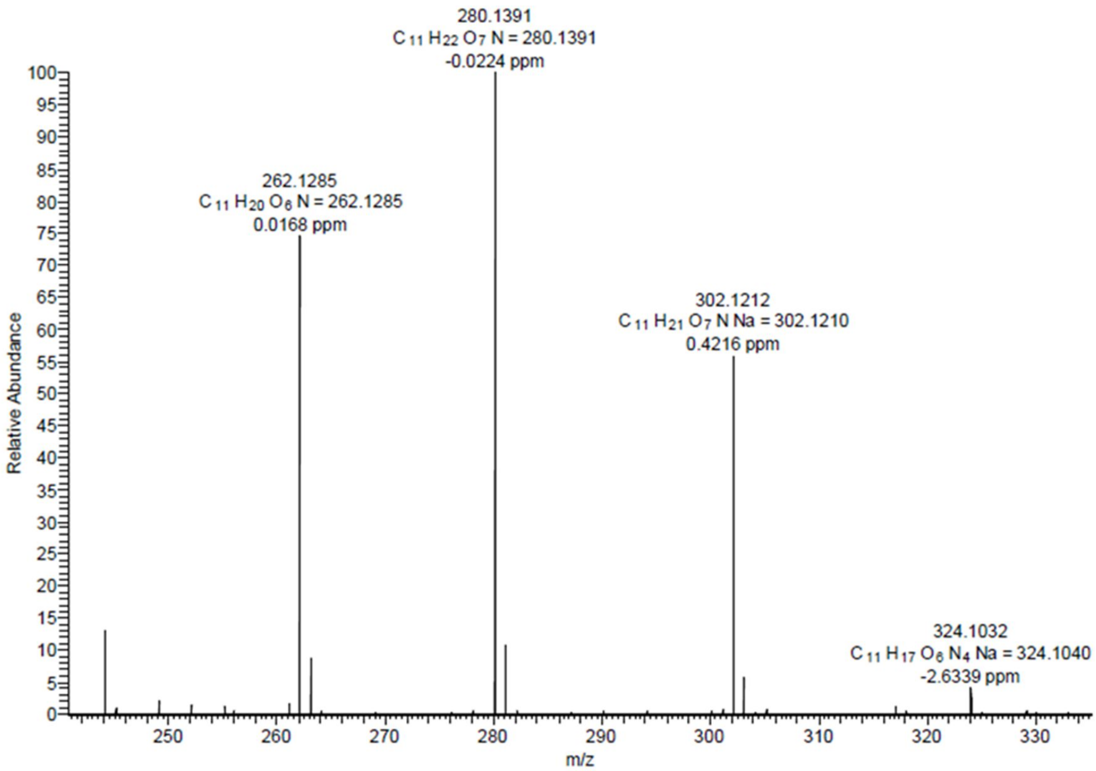

Fig. S27 HRMS (ESI^+ Orbitrap) spectrum of Flu-Val **4a**

Fructosylated Val-His (Fru-Val-His, 4b)

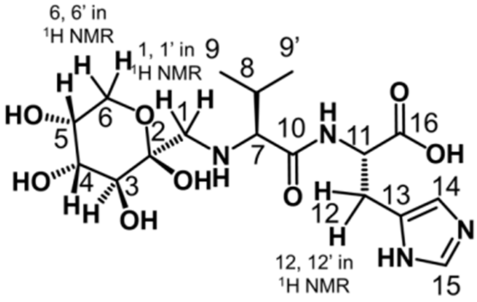

Table S8 Assigned signals from ¹H and ¹³C NMR spectra of Fru-Val-His **4b**, showing chemical shifts for both spectra as well as proton amounts, multiplicity and coupling constants for ¹H NMR

No.	¹H NMR				¹³C NMR
	Shift	Amount	Multiplicity	Coupling constant	Shift [a)]
1<br>1'	2.98 – 2.58	2	m	-	51.6
2	-	-	-	-	95.3
3	3.44 [b)]	1	m	-	70.46
4	4.03 – 3.96	1	m	-	83.2
5	3.83 – 3.75	1	m	-	75.0
6	3.61 – 3.48	1	m	-	60.0
6'	3.70 – 3.62	1	m	-	
7	3.95 – 3.91	1	m	-	64.6
8	2.28 – 2.17	1	m	-	29.1
9<br>9'	1.00 – 0.90	6	m	-	18.5 – 17.2
10	-	-	-	-	166.4
11	4.78 – 4.60	1	m	-	51.4
12	3.14 – 3.00	1	m	-	26.0
12'	3.32 – 3.15	1	m	-	
13	-	-	-	-	129.0
14	7.50 – 7.35	1	m	-	117.0
15	8.97	1	s	-	133.8
16	-	-	-	-	171.4
NH_2 (between 10 and 11)	9.01 – 8.99	1	m	-	-

a) most abundant ¹³C signal

b) below the overlying signal for residue ethanol

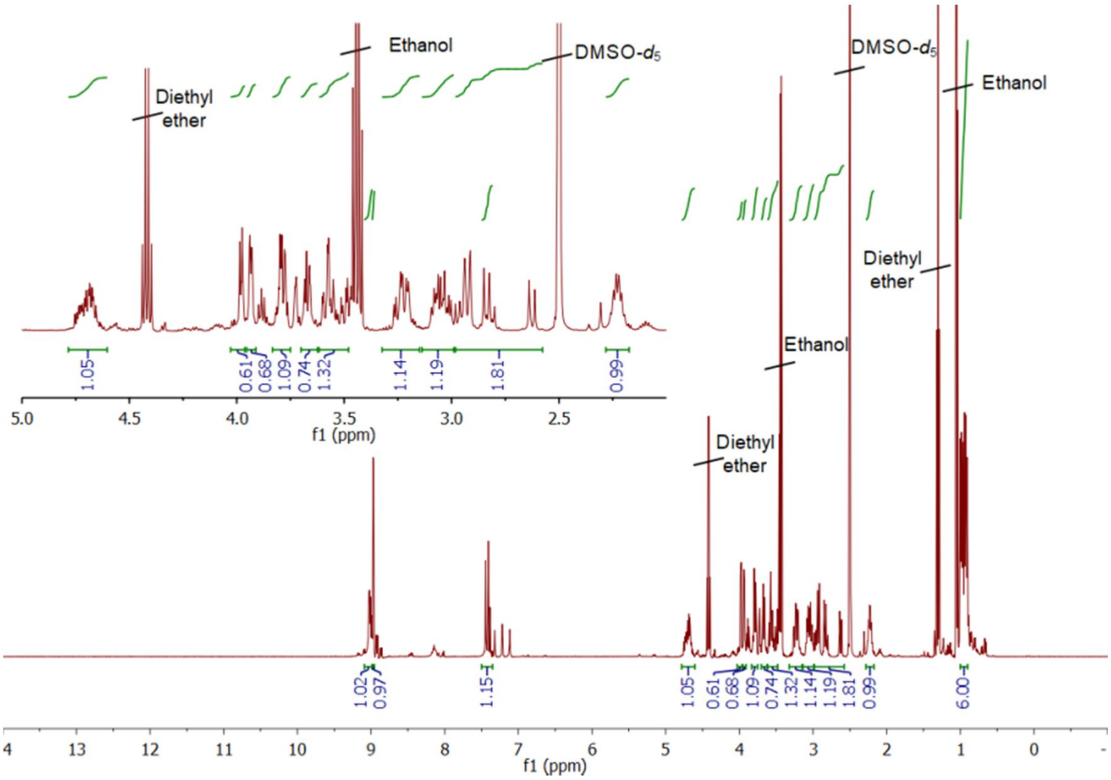

Fig. S28 ^{1}H NMR (500 MHz, DMSO-d_6) spectrum of Fru-Val-His **4b** with the enlarged area from δ 5.0 to 2.0 ppm

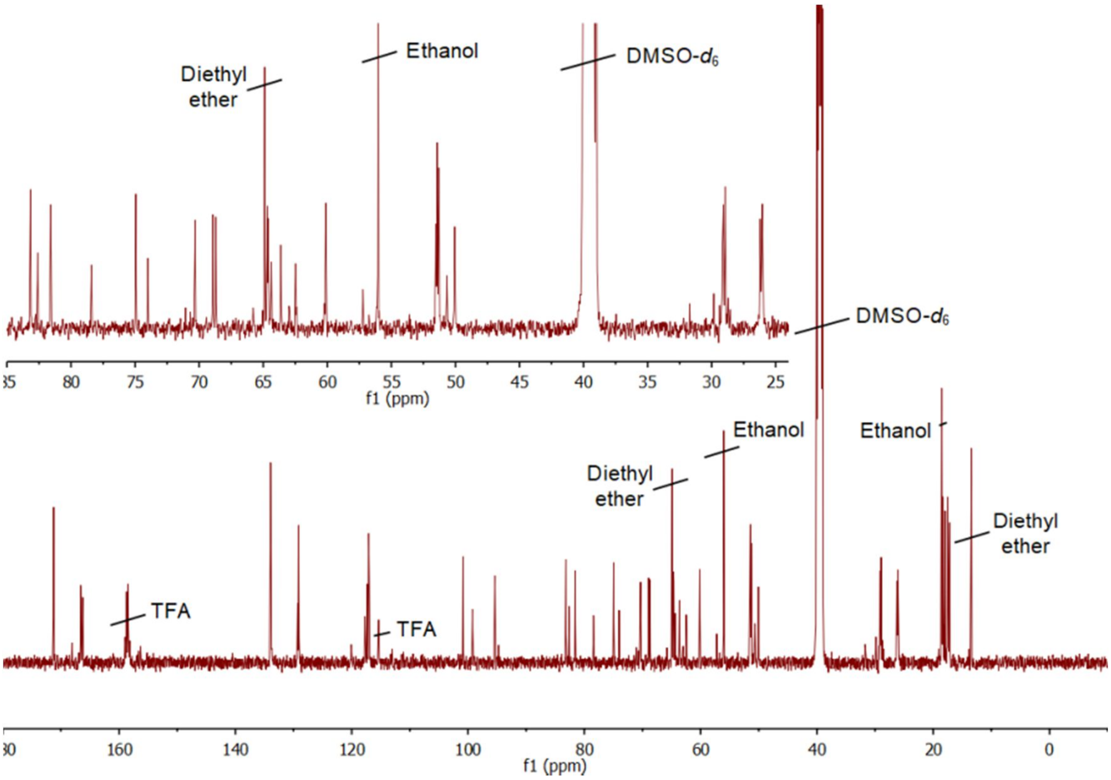

Fig. S29 ^{13}C NMR (126 MHz, DMSO-d_6) spectrum of Fru-Val-His **4b** with the enlarged area from δ 85 to 24 ppm

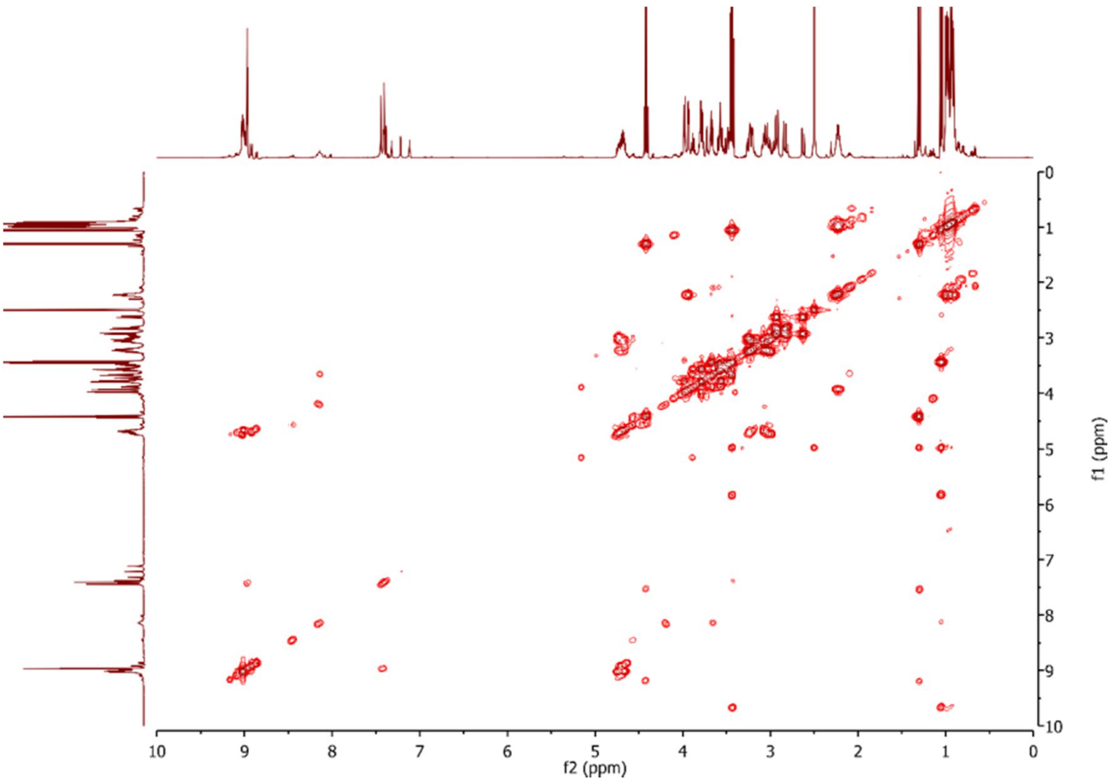

Fig. S30 ^{1}H-^{1}H-COSY spectrum of Fru-Val-His **4b**

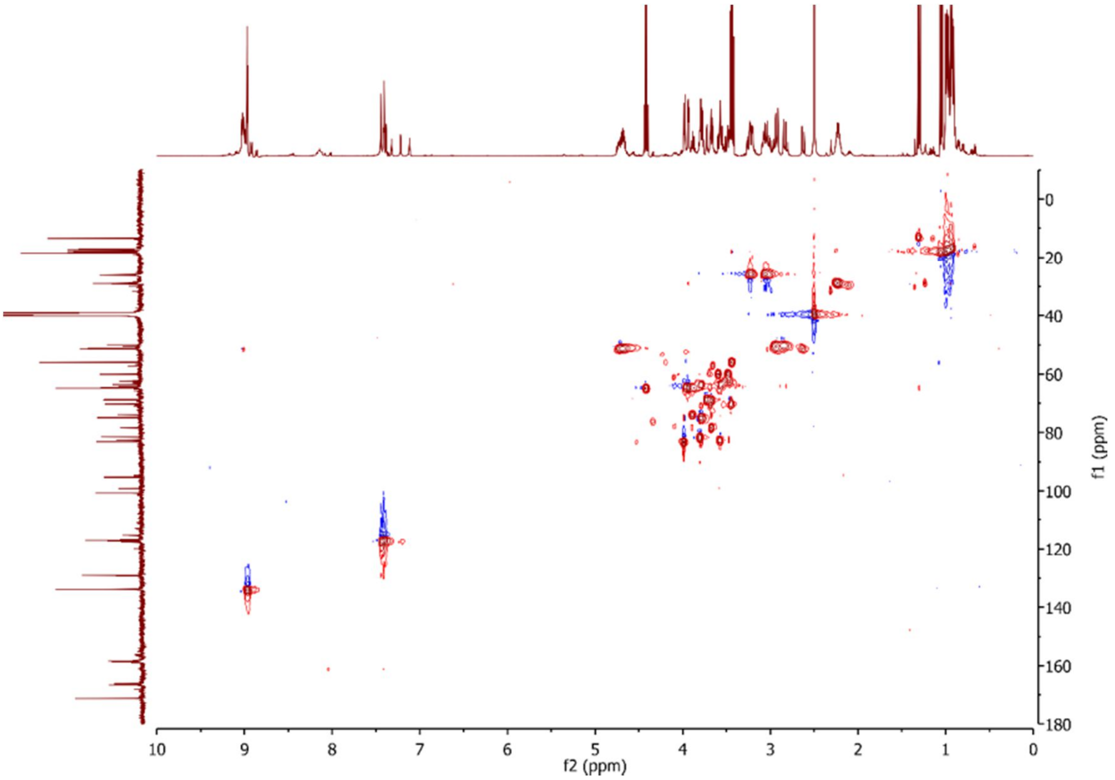

Fig. S31 ^{1}H-^{13}C-HSQC spectrum of Fru-Val-His **4b**

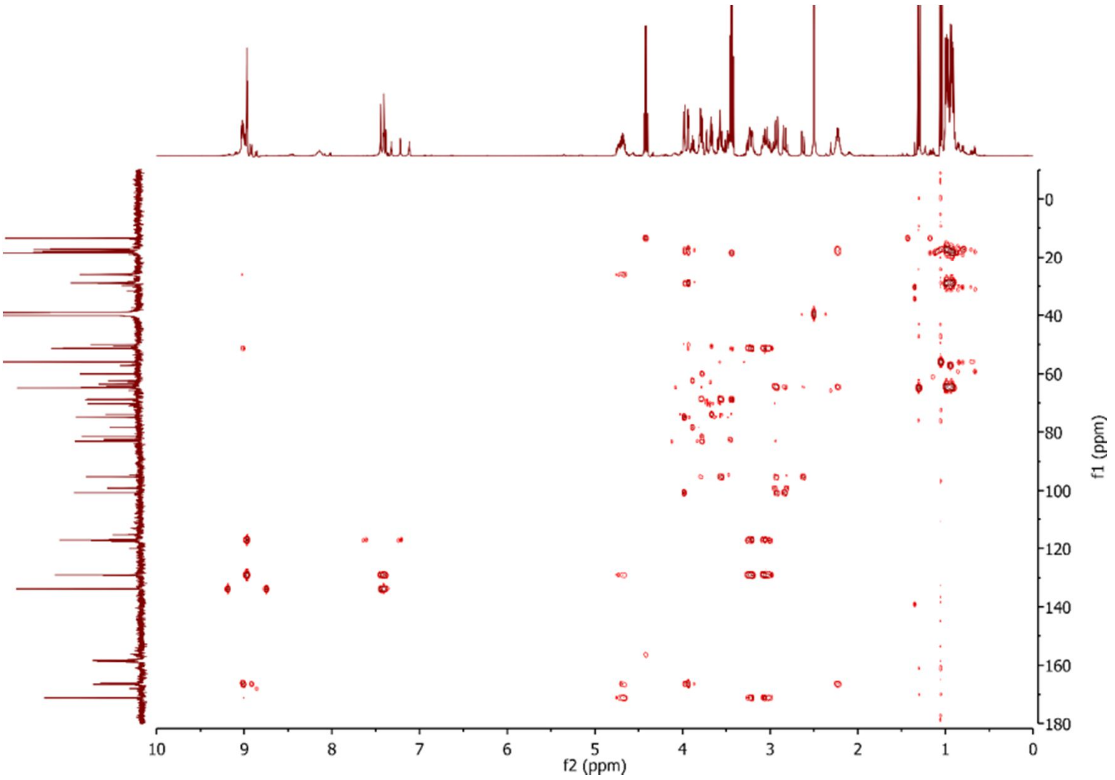

Fig. S32 ^{1}H-^{13}C-HMBC spectrum of Fru-Val-His **4b**

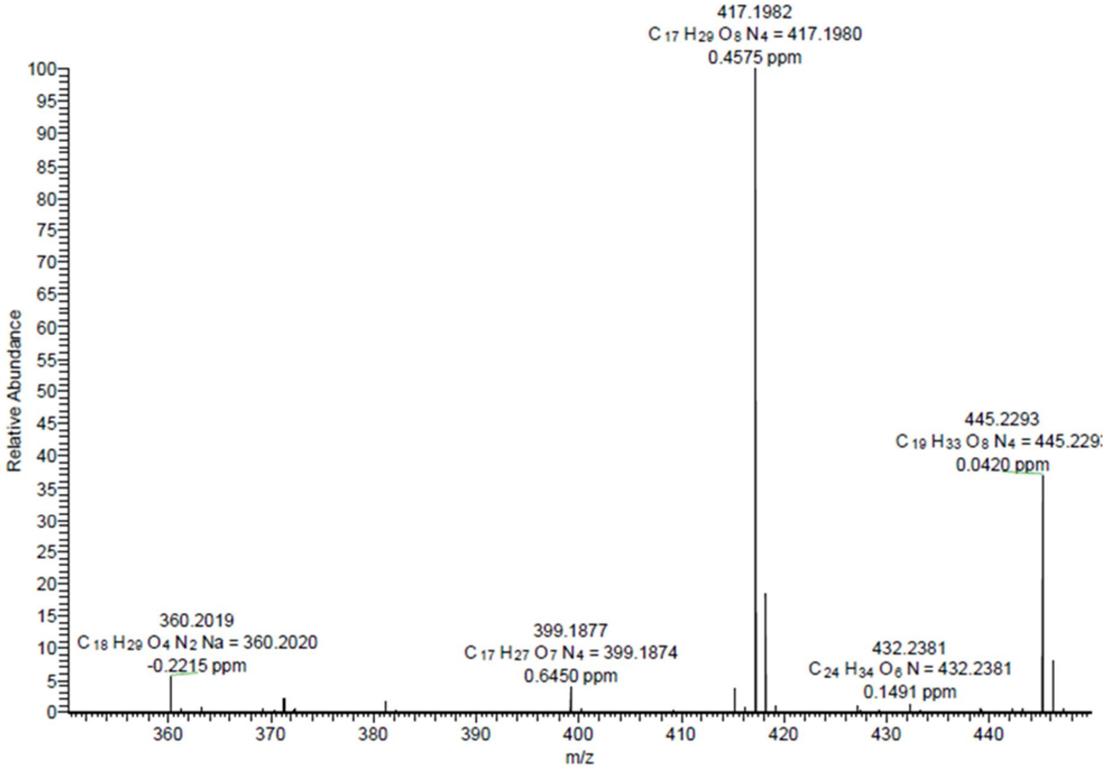

Fig. S33 HRMS (ESI^{+} Orbitrap) spectrum of Flu-Val-His **4b**

Proposed structures of the two most abundant transition ions

Amino acids and the dipeptide

Determined precursor ions for Val, His and Val-His were their monoprotonated adducts ($[M+H]^+$ with *m/z* of 118.0, 156.0 and 255.0 for Val, His and Val-His, respectively).

For the two amino acids Val and His the most abundant transition ions after CID are their immonium ions, as shown in **Fig. S34**, due to a cleavage of the carboxylic acid (monoisotopic mass loss of 46 u, caused by a neutral loss of carbon monoxide and water), which is a common fragment of amino acids in CID as stated in literature.[1] The *m/z* of the most abundant transition ions of Val and His as $[M\text{-}CO\text{-}H_2O+H]^+$ are 72.0 and 110.0, respectively. The second most abundant transition ions from the two amino acids have an *m/z* of 55.0 and 83.0 for Val and His, respectively, and their proposed structures are shown in **Fig. S34**.

For the dipeptide Val-His, the most abundant transition ion is caused by a fragmentation of the amide bond, resulting in the specific y_1 ion of Val-His, the monoprotonated His with an *m/z* of 156.0.[2] The second most abundant transition ion is again the immonium ion of His with an *m/z* of 110.0.

Possible structures of the precursor ions and the two most abundant transition ions of Val, His and Val-His are shown in **Fig. S34**.

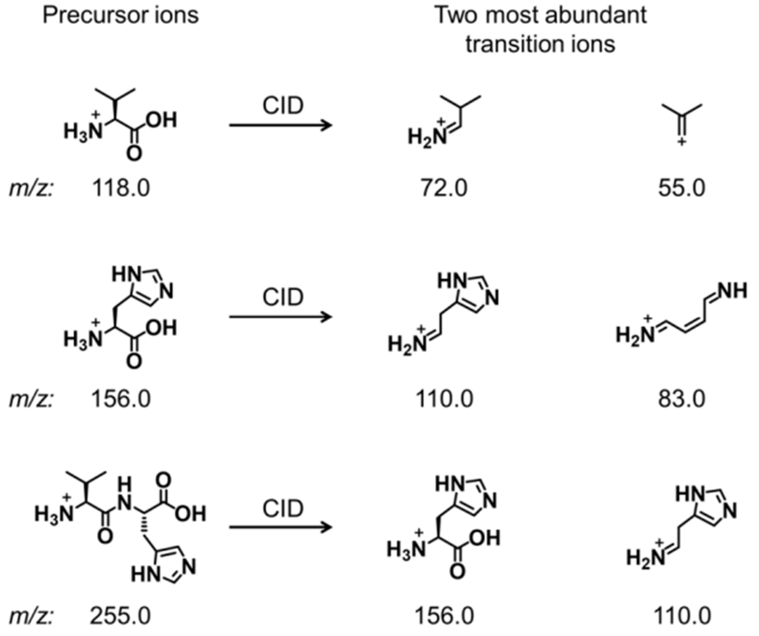

Fig. S34 Precursor ions and proposed structures of the two most abundant transition ions after CID of Val (top), His (center) and Val-His (bottom)

Protected sugars

Determined precursor ions for *ipr*Fru and *ipr*Glu (as *ipr*Glu-hydrate in aqueous solution) were their ammonium adducts ($[M+NH_4]^+$ with *m/z* of 278.1 and 294.0 for *ipr*Fru and *ipr*Glu-hydrate, respectively). During the fragmentation by CID, specific fragments of sugars were observed, although the alcohol groups are protected by isopropylidene groups. After the cleavage of the ammonium adduct and protecting groups, various neutral losses of water and formaldehyde were detected, resulting for both sugars in a variety of oxonium ions which are specific fragments for sugars as stated in the literature.[3]

The most abundant transition ions of *ipr*Fru after CID have *m/z* of 230.1 as well as 127.0 and of *ipr*Glu an *m/z* of 200.9 as well as 258.8 and their proposed structures are shown in **Fig. S35**.

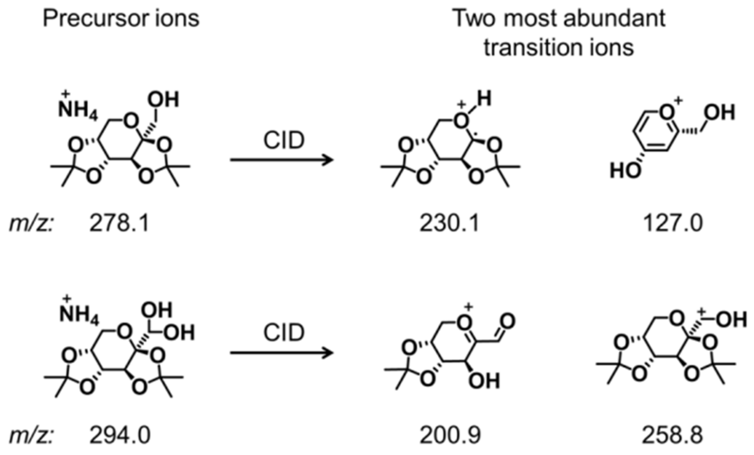

Fig. S35 Precursor ions and proposed structures of the two most abundant transition ions after CID of *ipr*Fru (top) and *ipr*Glu-hydrate (bottom)

iprFru-Val and Fru-Val

Determined precursor ions for iprFru-Val and Fru-Val were their monoprotonated adducts ($[M+H]^+$ with *m/z* of 360.0 and 280.1 for *ipr*Fru-Val and Fru-Val, respectively).

For both compounds, specific fragmentations of sugars as well as amino acids, common for fructosamines[4, 5], were observed and the two most abundant transition ions observed were very similar. The fragmentations of the sugar moieties are again of the protecting groups (for *ipr*Fru-Val) as well as neutral loss of water. Fragmentation on the amino acid residue leads for both compounds to the formation of the specific immonium ion.

The most abundant transition ions of *ipr*Fru-Val after CID have *m/z* of 302.2 as well as 256.1 and of Fru-Val an *m/z* of 262.0 as well as 216.0 and their proposed structures are shown in **Fig. S36**.

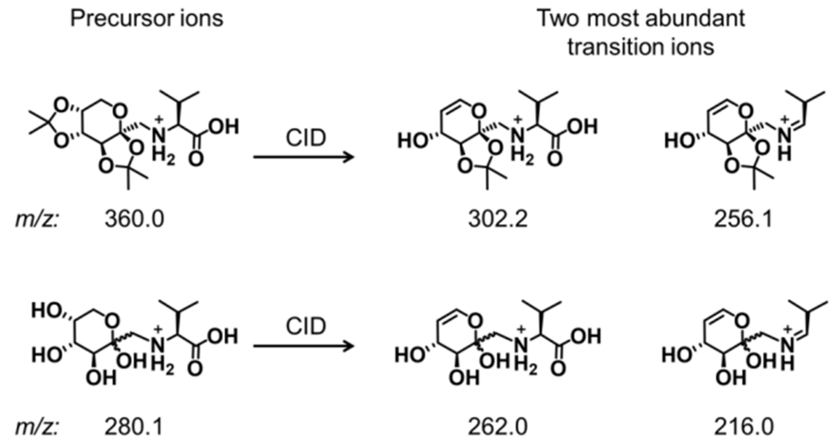

Fig. S36 Precursor ions and proposed structures of the two most abundant transition ions after CID of *ipr*Fru-Val (top) and Fru-Val (bottom)

iprFru-Val-His and Fru-Val-His

Determined precursor ions for *ipr*Fru-Val-His and Fru-Val-His were their monoprotonated adducts ($[M+H]^+$ with *m/z* of 497.2and 417.1 for *ipr*Fru-Val-his and Fru-Val-His, respectively).

For both compounds, the exact same fragments were observed, due to the formation of the specific y_1 ion of the dipeptide. The monoprotonated His subsequently further fragments to its immonium ion.

The most abundant transition ions of *ipr*Fru-Val-His and Fru-Val-His after CID have *m/z* of 156.0 as well as 110.0 and their proposed structures are shown in **Fig. S37**.

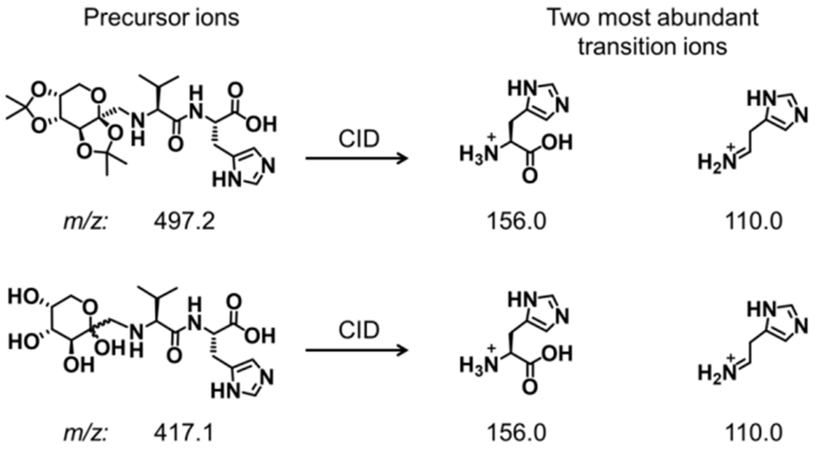

Fig. S37 Precursor ions and proposed structures of the two most abundant transition ions after CID of *ipr*Fru-Val-His (top) and Fru-Val-His (bottom)

Val-His quantification

The amount of remaining Val-His in synthesized Fru-Val-His was determined using the HILIC-ESI-MS/MS method. A five-point calibration curve of Val-His was measured to quantify remaining starting material in the final product. The five standards had concentrations of 0.775, 3.875, 7.75, 15.5 and 23.25 µg/mL. Obtained peak areas from the most abundant transition ion with an *m/z* of 156.0 were plotted against the concentrations of Val-His for each standard.

The calibration curve showed high linearity with a coefficient of determination (R^2) of 0.993. The calibration curve, the equation of the linear regression curve and R^2 are shown in **Fig. S38**.

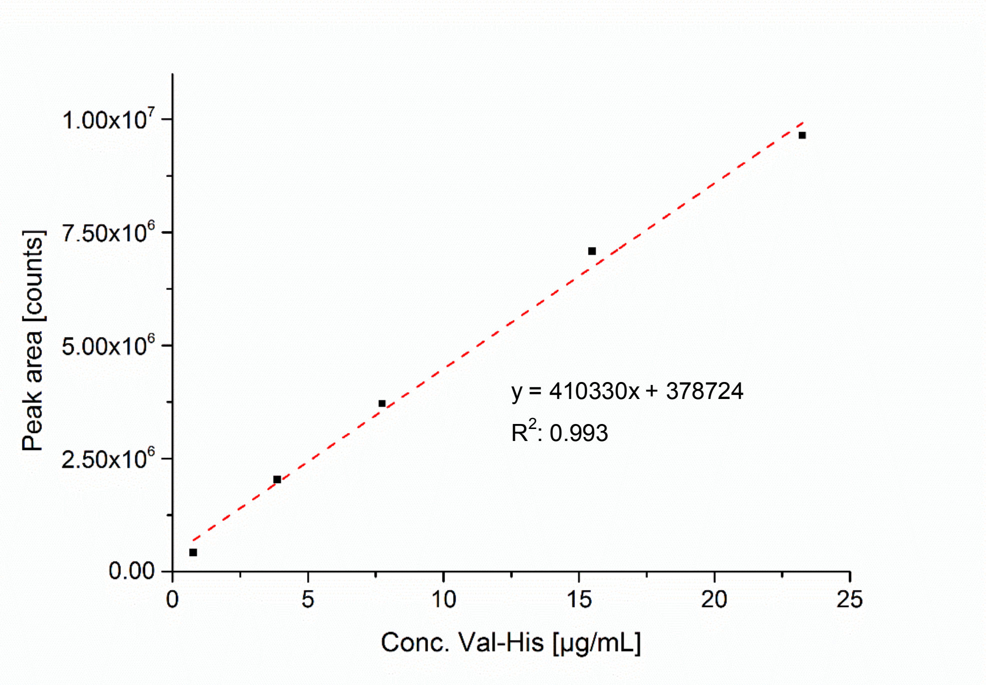

Fig. S38 Calibration curve of Val-His showing the linear regression curve for the plot of peak areas against the concentrations of Val-His with resulting equation and coefficient of determination R^2

For determination of Val-His in final Fru-Val-His, 138 µg/mL Fru-Val-His were dissolved in a mixture of ACN and H_2O (80/20, v/v) containing 5 mM NH_4HCO_2. A duplicate determination of the samples was performed. Peak areas from the two measurements were 2.24×10^6 and 2.29×10^6, which correspond to Val-His concentrations of 4.53 and 4.66 µg/mL, respectively. The 4.6 µg/mL (on average) correspond to 3% of the weighed in amount of sample.

References

1. Wang Y, Li S-M, He M-W. Fragmentation Characteristics and Utility of Immonium Ions for Peptide Identification by MALDI-TOF/TOF-Mass Spectrometry. Chin J Anal Chem. 2014;42(7):1010-6.
2. Seidler J, Zinn N, Boehm ME, Lehmann WD. De novo sequencing of peptides by MS/MS. Proteomics. 2010;10(4):634-49.
3. El-Aneed A, Banoub J, Koen-Alonso M, Boullanger P, Lafont D. Establishment of Mass Spectrometric Fingerprints of Novel Synthetic Cholesteryl Neoglycolipids: The Presence of a Unique C-Glycoside Species During Electrospray Ionization and During Collision-Induced Dissociation Tandem Mass Spectrometry. J Am Soc Mass Spectrom. 2007;18(2):294-310.
4. Frolov A, Hoffmann P, Hoffmann R. Fragmentation behavior of glycated peptides derived from D-glucose, D-fructose and D-ribose in tandem mass spectrometry. J Mass Spectrom. 2006;41(11):1459-69.
5. Mennella C, Visciano M, Napolitano A, Del Castillo MD, Fogliano V. Glycation of lysine-containing dipeptides. J Pept Sci. 2006;12(4):291-6.
